# A Multidimensional Definition of Pre-Osteoarthritis: Toward 21st-Century Subclinical Detection and Targeted Intervention

**DOI:** 10.3390/ijms262311447

**Published:** 2025-11-26

**Authors:** Eloy del Río

**Affiliations:** Independent Researcher, 11520 Cádiz, Spain; eloy.delrio@uca.es

**Keywords:** pre-disease/transition state/pre-osteoarthritis, early detection, phenotypes/endotypes/molecular subtypes, biomarkers, quantitative imaging, biomechanics, extracellular matrix, proteoglycan, aggrecanases, cartilage-protective strategies, osteoarthritis prevention/disease interception

## Abstract

Osteoarthritis (OA) is a leading cause of pain, disability, and healthcare utilization worldwide, yet clinical diagnosis commonly occurs after irreversible structural damage, limiting opportunities for prevention. Advances in molecular profiling, quantitative imaging, biomechanics, and longitudinal cohort studies have identified a reproducible preclinical interval, termed pre-osteoarthritis (pre-OA), during which molecular, compositional, and biomechanical perturbations emerge long before persistent symptoms or radiographic changes. The recognition of pre-OA as a distinct pathophysiologically meaningful stage supports the possibility of earlier targeted interception. Cross-disciplinary studies have consistently reported very early cartilage matrix alterations, pro-catabolic and low-grade inflammatory signatures, and biomechanical and biochemical marker shifts, indicating a critical detection window. Building on these findings, I propose a pheno-endotype-oriented framework to align emerging detection strategies with interventions matched to underlying mechanisms, including lifestyle modification, metabolic modulation, and candidate disease-modifying therapies. These conceptual models are presented for evaluation by clinicians, researchers, and healthcare decision-makers. Translation into practice remains constrained by heterogeneous case definitions, lack of validated thresholds, variability in assays and imaging standards, and limited prospective trials addressing early disease diagnosis. Addressing these barriers will require harmonized consensus criteria, standardized analytic protocols, prospective validation cohorts enriched with high-risk populations, and adaptive biomarker-driven trial designs. Reconceptualizing OA as a continuum with an identifiable preclinical stage provides a foundation for earlier personalized interception strategies with the potential to alter the natural history of the disease and reduce its global burden. If translated successfully, early identification and targeted interception of pre-OA could transform OA from an inevitable consequence of aging into a largely preventable and manageable condition, which would be a paradigm shift with major clinical and public health implications.

## 1. Background, Rationale and Objectives

Osteoarthritis (OA) affects approximately 595 million people worldwide and remains a major public health challenge [1]. Despite substantial advances in our mechanistic understanding of this complex and disabling condition, clinically validated disease-modifying interventions are largely lacking in routine practice, and severe disease commonly culminates in total joint arthroplasty—an outcome that underscores the irreversible consequences of late-stage OA and motivates primary prevention [2]. Etiologically, OA is a multifactorial, heterogeneous disease driven by genetic, biomechanical, metabolic–inflammatory, and environmental factors, whose interactions at both the systemic and joint levels determine disease initiation and progression. Importantly, these interacting influences often produce a prolonged subclinical phase during which pathobiological changes occur long preceding the onset of symptoms or radiographic signs [3,4,5,6,7,8,9,10,11,12,13]. This temporal disconnect gives rise to the “cracked mirror” problem [14]: traditional symptomatic and imaging-based outcome measures—often used as benchmarks for biomarker evaluation, that is, the mirror against which biomarkers are typically validated—may not reflect the same pathobiological pathways or time courses captured by molecular markers. Reliance on these outcomes can therefore impede biomarker qualification and the identification of true biological endotypes. Notably, recent studies have demonstrated that panels of circulating molecules can predict incident or progressive knee OA years before radiographic abnormalities or overt symptoms, highlighting both the promise and validation challenge for systemic biomarkers [15]. When OA becomes clinically apparent, structural damage, altered mechanics, chronic inflammation, and systemic comorbidities are typically established, creating feedback loops that confound causal inferences and limit the translational value of late-stage mechanistic models. Modeling therefore faces a trade-off: highly realistic multiscale approaches capture biological complexity but are difficult to parameterize and validate, whereas simpler models sacrifice mechanistic detail for tractability [16,17]. Together, these overlapping limitations underscore the need to focus on the subclinical phase of OA, where early molecular and cellular perturbations can be measured and modeled. By defining a multidimensional “pre-osteoarthritis” (pre-OA) state, researchers can capture ultra-early signals, facilitate feasible modeling, and design individualized early intervention trials that bridge the gap between mechanistic insights and clinically meaningful outcomes.

The pre-OA state exhibits a relatively circumscribed set of ultra-early cellular, matrix, inflammatory, and biomechanical perturbations that are readily simplified conceptually and measured empirically, making it an ideal substrate for developing mathematical, omics-based, computational, and mechanistic models that balance interpretability and biological fidelity [18]. Conceiving OA as a continuum rather than an isolated end-stage entity reframes these ultra-early changes as measurable signals instead of inscrutable end points, thereby opening a window for modeling that links molecular and cellular dysregulation with tissue-level trajectories and individualized risk profiles [5,6,8,19,20,21,22]. However, the drivers and modulators of this subclinical phase remain poorly understood. Simultaneously, advances in longitudinal phenotyping, high-resolution compositional imaging, synovial and molecular biomarker assays (e.g., proteomics and metabolomics), high-throughput cellular assays, and continuous biomechanical monitoring (e.g., wearable gait/load sensors) have made rigorous model calibration and prospective validation against real-world trajectories increasingly feasible [4,23,24,25]. These convergent developments strengthen our ability to map mechanistic signals to tissue and individual outcomes and design risk-stratified early intervention paradigms, adaptive stepped-care algorithms, and combination therapies that target mechanical and biological drivers in parallel. Together, these trends suggest that a tangible path toward a predictive, experimentally grounded theory of pre-OA is realistically achievable, provided that model development is coupled with standardized measurement protocols, suitable longitudinal cohorts for external validation, and pragmatic trial designs that employ validated subclinical markers for enrichment and intermediate endpoints.

This article presents no new primary data; rather, it synthesizes recent conceptual and technological advances to propose a pragmatic, testable framework for defining and operationalizing pre-OA. The aim is to catalyze coordinated initiatives across basic research, clinical investigation, implementation science, regulators, and policymakers to validate the framework, establish thresholds, and translate subclinical detection into equitable early-intervention strategies that reduce the population burden of OA in the coming decades [2]. Key knowledge gaps include definitive biomarker thresholds, harmonized quantitative imaging protocols, validated wearable and gait metrics, interoperable and adequately powered longitudinal cohorts that reflect demographic and geographic diversity, and evidence of the real-world feasibility, cost-effectiveness, and equity of screening and intervention pathways [4,14,26]. To address these gaps, I recommend a phased research program: (1) multi-stakeholder consensus panels to define minimal measurement sets and analytic standards; (2) prospective validation studies and coordinated cohort initiatives to calibrate models against clinical trajectories; (3) development of secure, interoperable data repositories with standardized data dictionaries and governance for reproducible model development; and (4) phased pilot implementation trials that use validated subclinical biomarkers (e.g., soluble peptides, genomic and epigenetic signatures, microRNAs, and imaging markers) for cohort enrichment and as intermediate endpoints, coupled with formal assessments of cost-effectiveness and differential access [14,27]. Attention to feasibility, economic value, and equitable reach should be embedded throughout, with study designs that both explicitly assess performance across under-represented populations and address the limitations of the “cracked mirror” by prioritizing temporally appropriate endpoints, molecularly informed analytic frameworks, and intermediate subclinical outcomes that reflect underlying biology more accurately [14]. The qualitative multidimensional models presented herein are intended to guide clinicians, researchers, and policymakers by highlighting the interplay among the biological, mechanical, and environmental factors that drive OA development and fostering multidisciplinary dialogue. The following sections describe the proposed models, provide exemplar measurement tools for each domain, and outline the next steps for validation and clinical translation. By characterizing pre-OA as a measurable, multidimensional construct and aligning modeling with standardized measurements and validation pathways, the field can shift from reactive management to proactive, evidence-based, ultra-early intervention.

## 2. Pre-OA as a Distinct Pre-Disease State: Insights from Pre-Diabetes and Pre-Hypertension

The concept of pre-disease states is central to preventive medicine. Generally, the prefix “pre-” denotes a clinically measurable intermediate state between health and established disease that identifies an increased risk before the presence of diagnostic pathology [28,29,30,31]. Well-recognized examples include prediabetes and, historically, prehypertensive states. Prediabetes (impaired fasting glucose or HbA1c 5.7–6.4%) is predictive of an elevated likelihood of progression to type 2 diabetes and—importantly—has evidence-based interventions (intensive lifestyle modification; selected pharmacotherapy) that reduce the risk of progression [32,33]. Prehypertension, traditionally defined as systolic 120–139 mmHg or diastolic 80–89 mmHg, similarly represents a measurable “gray zone” associated with elevated cardiovascular risk, although blood pressure thresholds and guideline recommendations have evolved over time [34]. These “pre-” states share several key features: objective measurability, subthreshold pathology, prognostic value for progression, and the existence of interventions that can prevent or delay disease. From this perspective, Viera [28] proposed that a pre-disease state is clinically meaningful when it identifies individuals at significantly increased risk of developing overt disease and when effective interventions exist that can modify, attenuate, or slow progression. Applying this rubric to OA implies that pre-OA could be considered a valid pre-disease category if early structural, molecular, or biomechanical indicators reliably predict symptomatic OA, and if targeted interventions can modify the disease trajectory. Accordingly, the identification and longitudinal validation of such putative early indicators should be prioritized to enable earlier and scalable detection.

**Figure 1:**
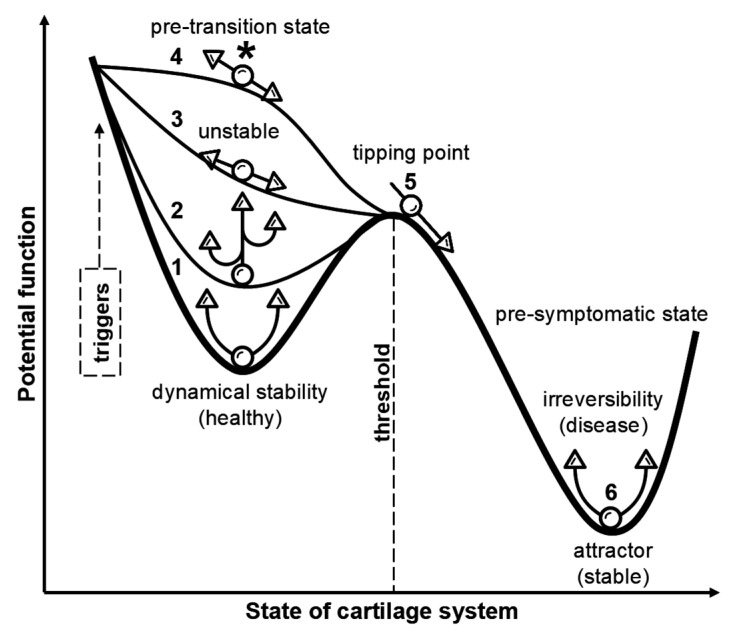
Conceptual potential energy landscape illustrating the transition from a homeostatic molecular baseline through an intermediate pre-disease state to early OA. The schematic maps the cartilage system onto a qualitative potential-like surface: the horizontal axis represents joint state (left = healthy; right = established OA) and the vertical axis represents a quasi-potential that encodes resilience (low = deep, stable basin; high = unstable). Discrete basins (attractors) and saddles (barriers) illustrate multistability and the susceptibility of the system to be driven between regimes. The dashed arrow indicates representative perturbations (mechanical overload, metabolic dysregulation, systemic inflammation, and hormonal change) that alter basin depth or lower barriers and hence increase the transition probability. The figure highlights core dynamical concepts—positive (self-reinforcing) versus negative (damping) feedbacks, tipping points/unstable equilibria, and early-warning indicators (e.g., increased variance, slowed recovery). Numbered callouts indicate schematic stages: (1) healthy: deep, narrow basin; low quasi-potential; high resilience and rapid recovery; (2) pre-OA: shallower, broader, metastable basin with higher quasi-potential and reduced resilience; (3) critical period: landscape flattens and the system nears a saddle (unstable equilibrium); early-warning signals (increased variance, critical slowing) may emerge; (4) escalation: saddle lowered by persistent insults further lowers the barrier and increases transition probability/risk; (5) transition: bifurcation/tipping point; the healthy attractor disappears and the system rapidly moves into the disease basin; change can be abrupt and partly irreversible; (6) early OA: established disease attractor stabilized by self-reinforcing pathology; self-sustaining pathology; high barrier to full recovery. This qualitative conceptual model provides a rationale for early, multimodal detection and stratified interventions. Adapted from [36].

To make the pre-disease concept accessible, Figure 1 theoretically depicts the OA continuum as a qualitative quasi-potential “landscape” that maps joint cartilage system states onto attractor basins and energy barriers, illustrating resilience, metastability, and tipping-point transitions [35]. Specifically, the healthy state (1) occupies a deep, narrow valley—low potential energy, rapid recovery from physiological perturbations, and robust homeostatic repair. A distinct pre-OA basin (2) lies slightly “uphill”: shallower and broader, with higher potential and reduced resilience, such that repeated or cumulative stresses produce larger perturbations and slower recovery. Subtle compositional MRI changes and early shifts in matrix biomarkers likely emerge here. As adverse loading or systemic stress accumulates, the landscape flattens (3), signaling a critical period marked by early-warning signals such as increased variance and delayed recovery. Continued parameter drift (4)—from obesity, chronic overload, or hormonal perturbations—lowers the barrier and increases the probability of crossing into disease. The transition (5) can be abrupt: an imminent bifurcation where the healthy attractor collapses and the system descends into the early OA basin (or drastic deterioration). Once this attractor (6) is established, pathological positive feedback loops involving matrix breakdown, low-grade inflammation, and subchondral remodeling reinforce degradation and tend to self-perpetuate themselves. Additionally, the barrier to full restoration is high, and persistent compositional defects, intermittent pain episodes, and sustained degradative biomarker levels become evident. This landscape metaphor also emphasizes that disease progression is not defined solely by static tissue loss and purely structural processes but by dynamic system resilience and barrier thresholds—features that inform the timing and selection of early, pre-emptive, mechanism-matched interventions that can guide multimodal biomarker panels and stratified trial designs.

Conceptually, the term pre-OA has emerged to describe the at-risk early stage of joint degeneration [6,8,18,20,37,38,39,40,41,42,43,44]. Analogous to other pre-disease states, pre-OA emphasizes a window for preventive and disease-modifying strategies, including weight management, targeted exercise, biomechanical correction, and emerging pharmacologic therapies, which have the potential to alter disease trajectories. Yet, unlike prediabetes or prehypertension, pre-OA remains largely a research construct, and standardized clinical criteria and universally validated biomarkers are lacking. Current initiatives, such as those led by the Osteoarthritis Research Society International (OARSI) [45], aim to define early stage OA and create a consensus on measurement standards, setting the stage for a paradigm shift from reactive care to proactive interception. Quantitative and compositional imaging (e.g., advanced MRI techniques) and candidate molecular biomarkers of cartilage turnover and low-grade inflammation are under active investigation; however, none have yet been incorporated into routine clinical practice. Implementation will require not only bioanalytic validation but also a formal evaluation of net clinical benefit, feasibility, and equity.

Similar to metabolic and cardiovascular diseases, OA progresses along a prolonged, multifactorial structural–clinical continuum characterized by cartilage matrix degradation, subchondral bone remodeling, synovial inflammation, and altered joint biomechanics. Notably, the earliest clinically measurable signals in OA are typically multimodal—molecular, compositional/structural (tissue), and biomechanical—rather than a single, widely available systemic biomarker [4,14,15,23,24,25,26,27,40,44,46]. For example, compositional MRI (T2/T1ρ/dGEMRIC), targeted synovial, serum/plasma (e.g., sCOMP, sHA), or urine (e.g., uCTX-II) proteomic markers, and biomechanical measures (peak knee adduction moment or wearable-derived cumulative loading) capture the complementary aspects of early joint changes. In contrast to prediabetes or elevated blood pressure, where reproducible numeric thresholds enable population screening, routine detection of pre-OA currently depends on specialized, heterogeneous tools that lack standardized benchmarks and broad availability [28,29,30,31,32,33,34,35,36]. Consequently, translating the OA continuum into a pragmatic “pre-disease” category will require harmonized multimodal criteria that combine clinical risk profiling with standardized imaging, biomarkers, and biomechanical measures—for example, contact pressure, compressive strain, fibril strain, and maximum shear strain—together with rigorous analytic and prospective validation to define predictive thresholds and scalable, inclusivity-oriented deployment strategies [17,21,44].

Clinical diagnosis of OA—based on pain, stiffness, and radiographic changes—usually occurs after substantial, often irreversible, joint damage. Emerging evidence, however, highlights earlier subclinical alterations, including extracellular matrix (ECM) molecular changes, compositional cartilage abnormalities, focal tissue loss, MRI-detectable defects, bone marrow lesions (BMLs), early biomechanical dysfunction, accompanied by chondrocyte phenotypic shifts, dysregulated mechanotransduction, and early catabolic signaling, all of which precede symptomatic OA development [3,4,5,6,7,9,10,11,12,13,23,25]. These ultra-early signals remain challenging to measure, standardize, and translate into routine care, particularly in asymptomatic individuals. In a recent proof-of-concept study, Oshima et al. [23] combined Raman spectroscopy with dynamic network biomarkers (DNBs) to identify “transition states” between health and disease, demonstrating that cartilage compositional fingerprints can detect early degenerative changes. Specific Raman bands—approximately 1042 cm^−1^ (C–O–C stretching) and 1061 cm^−1^ (O–SO_3_^−^ vibrations)—were associated with glycosaminoglycan content and represent candidate molecular markers for pre-OA. Although methodologically promising, these approaches require disease-specific analytic validation, reproducibility testing across centers, prospective prognostic studies, and demonstration of clinical utility and accessibility before clinical deployment [23,25,47].

Overall, predisease states are valuable when they enable targeted interventions that demonstrably reduce future disease burden, providing a rationale for their use in preventive healthcare [28]. Prediabetes and—classically—prehypertension are established clinical categories with standardized definitions that facilitate predictive population-level risk stratification and early intervention. Although conceptually analogous, pre-OA remains unstandardized and primarily research-based, lacking standardized criteria and universally validated biomarkers [8,18,20,40,44]. The growing recognition of pre-OA signals an evolving paradigm in OA research and care, in which identifying and intervening during the earliest stages could prevent or delay symptomatic, radiographically evident disease, provided that reproducible risk-stratification criteria are established and robust interventional evidence demonstrates a favorable net benefit. Any implementation strategy must also address potential harms (overdiagnosis, inequitable access) and demonstrate feasibility and cost-effectiveness in diverse populations. The following sections review candidate markers, measurement standards, and trial designs to advance this agenda.

## 3. Structural and Functional Complexity of Articular Cartilage: Implications for Tissue Regeneration and OA Management

Hyaline cartilage is essential for joint function, providing a low-friction articulating surface and absorbing mechanical loads to preserve mobility and joint integrity [48,49]. Its function derives from a highly integrated, few-millimeter-thick ECM in which type II collagen, aggrecan-rich proteoglycans, and surface lubricants (e.g., PRG4/lubricin) combine with a sparse population of chondrocytes to produce exceptional load-bearing and tribological properties. This intimate coordination of biophysical and biochemical traits makes cartilage a paradigmatic example of form–function coupling in musculoskeletal biology, but also creates a translational paradox: the intrinsically limited reparative capacity of cartilage renders it vulnerable to progressive, often irreversible damage [50]. Structural degradation of the ECM—comprising collagen network disruption, loss of glycosaminoglycan-mediated water retention, and altered viscoelasticity—substantially impairs cartilage mechanical performance. Despite decades of research in tissue engineering and regenerative medicine, restoring functional joint cartilage remains challenging.

To capture its extraordinary nature, Cederlund and Aspden [51] aptly remind us that *articular cartilage has the water content of a banana, a compressive modulus similar to silicone rubber, and is as impermeable as granite. It often provides a bearing surface with a friction coefficient akin to ball bearings, lasting for a lifetime. The question remains: how does it achieve this remarkable combination of properties?* Importantly, this unique interplay between structural and mechanical traits represents a paradox at the crossroads of biology and mechanics, underscoring the complexity of developing effective OA treatment strategies. Restoring functional joint cartilage therefore requires re-establishing a depth-dependent zonal architecture (a lubricating superficial zone overlying a load-bearing deep zone), recreating a competent osteochondral interface, including the tidemark, and preventing inappropriate hypertrophy or aberrant endochondral ossification [21,52,53,54]. Although tissue engineering strategies (zonal scaffolds, osteochondral grafts, cell-based implants, and mechanically conditioned hydrogels) show promise, major challenges remain in reproducing the native microarchitecture, achieving durable mechanical integration with the subchondral bone, and restoring lifelong low-friction function. These constraints underscore why early, pre-emptive interventions in pre-OA—when defects are compositional or focal rather than extensive—offer the best chance for successful tissue preservation or regeneration.

Replicating the native zonal architecture remains a key barrier to durable cartilage regeneration. Professional societies, including the American Academy of Orthopaedic Surgeons (AAOS), have raised concerns about the premature clinical adoption of biological therapies, such as platelet-rich plasma (PRP) and unvalidated cell-based treatments, in the absence of rigorous evidence and appropriate regulatory oversight [55,56,57]. This trend is driven by high patient demand, limited conventional treatment options, and aggressive direct-to-consumer marketing of unproven interventions that bypass standard regulatory oversight. The spread of misinformation risks damaging public trust and impedes the careful development of validated biological therapies [57]. Native hyaline cartilage, an evolutionary masterpiece of natural engineering, acquires complex, depth-dependent zonal organization and functional maturation over the years, and engineering a durable replacement therefore requires precise cellular colonization, ECM assembly, and faithful reconstruction of the cartilage–bone interface [58]. Given that the superficial and deep zones differ markedly in biochemical composition and mechanical role (lubrication versus compressive load-bearing), current regenerative techniques face significant barriers in fully restoring zonal integrity and long-term function [58,59,60,61]. Addressing these multidimensional challenges requires not only improved engineering solutions (zonal scaffolds, osteochondral constructs, and targeted cell- or matrix-based therapies) but also robust clinical evidence—randomized trials with long-term structural and functional endpoints, registries, and post-market surveillance—to demonstrate safety, efficacy, cost-effectiveness, and equitable access. While definitive cures for OA remain elusive, early detection and intervention, informed by deeper cartilage biology and rigorous evaluation, provide promising directions for preserving joint function.

## 4. Challenging Misconceptions and Enhancing Awareness for Early OA Prevention

OA has been relatively neglected in research and public health priorities, contributing to conceptual fragmentation and inconsistent definitions across disciplines—rheumatology, orthopaedics, radiology, physiotherapy, and basic science—an issue clearly highlighted by recent Delphi panels [62], which documented wide disagreement on key OA definitions and staging criteria. The gradual, nonlinear course of the disease complicates the association of early signs with a single clinical explanation, fostering persistent myths that impede accurate understanding and timely management (for example, “OA is an inevitable consequence of ageing”, “rest is best”, and “radiographic change is required for diagnosis”) [63,64]. Such misconceptions delay preventive action and can worsen outcomes.

**Figure 2:**
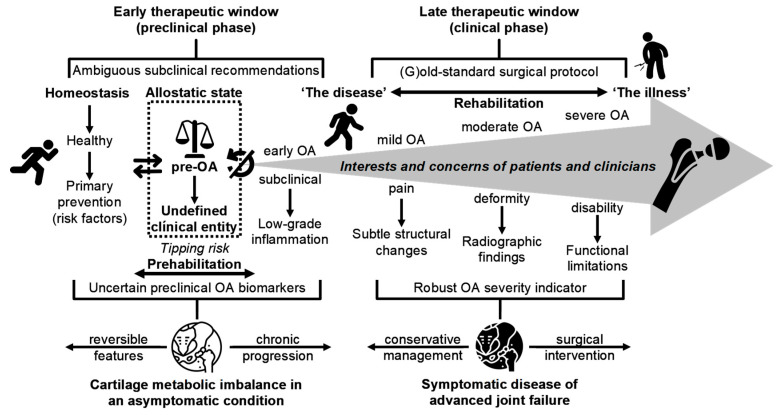
OA as a continuous spectrum: development, progression, diagnostic challenges, and early intervention opportunities. The schematic maps joint states from an asymptomatic, cartilage-confined pre-OA phase through intermediate tissue changes to established radiographic OA. Molecular and compositional alterations (detectable by molecular imaging, compositional MRI, and emerging biomarker assays) typically precede JSN and osteophyte formation visible on plain radiography, creating a diagnostic gap and critical window for early intervention. Accumulation of modifiable “tipping risks” (for example, prior joint injury, chronic abnormal loading or malalignment, obesity/metabolic dysfunction) can lower system resilience and precipitate a rapid transition to symptomatic disease; individual thresholds vary. The figure highlights the measurement and intervention priorities across the continuum (prevention, early detection, mechanism-matched therapy) and encodes the progression visually (color gradient and arrow). The arrow (small → large) denotes progression from an asymptomatic early stage to clinical OA, with increasing thickness reflecting rising therapeutic priority; its absence in the cartilage-confined pre-OA zone marks the early gap before overt multi-tissue disease.

Current evidence suggests that OA is an insidious, cumulative process rather than a single, abrupt event, often clinically silent until later stages, with early molecular and biomechanical changes preceding symptom onset and limiting the efficacy of late-stage interventions. As illustrated in Figure 2 and summarized in Table 1, cartilage damage and joint degeneration begin subtly and can advance significantly before symptoms become noticeable. This figure highlights the significant gap in the initial detection, where current diagnostic methods may miss critical early changes, thus focusing more on identifying risk factors for progression rather than true disease initiation. Although radiographic changes may eventually reveal the presence of OA, they often reflect late-stage disease, making them more suitable for monitoring progression rather than early detection [65]. This limitation underscores a substantial gap in the timely preclinical recognition, as current methods may overlook critical early changes, focusing primarily on identifying risk factors for disease progression rather than the earliest pathophysiological onset of OA. Herrero-Manley et al. [66] recently emphasized that current diagnostic criteria for early knee OA—which rely primarily on self-reported symptoms, imaging, and clinical findings—may lack accuracy and increase the risk of misdiagnosis, given that the manifestation of “symptoms” like joint pain and “signs” such as joint stiffness and mechanical sensitivity can be inconsistent and context-dependent. Although molecular biomarkers and advanced imaging approaches show promise for earlier detection and mechanistic insight [4,14,15,26,67,68], their large-scale implementation is currently constrained by complexity, cost, and a lack of standardized measures, underscoring the urgent need for improved diagnostic approaches that can effectively identify and address OA before significant irreversible damage occurs.

Historically described as “degenerative arthritis” or “osteoarthrosis”, OA was viewed primarily as inevitable mechanical *wear and tear* associated with aging. Early usage even applied the term “preosteoarthrosis” to individuals with mild, intermittent, activity-related knee pain perceived to be at risk of overt disease [86,87]. Yet, there is a broad consensus that OA is fundamentally a biological process rather than solely mechanical. Contemporary evidence has redefined OA as a biologically driven disorder in which low-grade, innate immune–mediated inflammation at the synovium–cartilage interface interacts with biomechanical and metabolic factors to drive joint degeneration. This reframing—from “osteoarthrosis” to “osteoarthritis”—emphasizes the role of autocrine and paracrine signaling, distinctive cytokine milieus, and tissue-specific degradative pathways that are mechanistically distinct from the systemic autoimmune inflammation of rheumatoid arthritis (RA) [88,89,90,91,92]. Importantly, OA is not synonymous with chronological aging; phenotypes and mechanistic endotypes reflect differences in molecular aging processes—including the emergence of pre-senescent and fully senescent cells, altered proteostasis, and inflammaging—that are modulated by organ vulnerabilities and lifestyle exposures and vary in pace and pattern across individuals (a concept sometimes framed as “dynamical aging”) [21,29,30,31,36,90,93,94]. This conceptual shift has practical implications: viewing OA as a biologically mediated, heterogeneous disorder driven by variable rates of molecular aging (rather than an inevitable consequence of chronological age) strengthens the rationale for developing predictive biomarkers and mechanism-targeted early intervention strategies aimed at preventing irreversible joint damage.

Articular cartilage has a limited intrinsic capacity for repair [50]; consequently, its degradation represents a critical initiating event that drives OA progression. By the time the condition is clinically or radiographically recognized, the therapeutic window for effective disease-modifying intervention is frequently lost, allowing irreversible progression to symptomatic disease with substantial pain and functional limitation. Biochemical and ultrastructural changes in cartilage, including proteoglycan depletion, loss of aggrecan-mediated hydration, and collagen network disruption, can precede clinical signs by months to years, creating a major translational gap between pathobiology and care. Although structural deterioration generally accelerates with age, the degenerative process is typically insidious and often subclinical, underscoring the need for early detection and proactive management. Integrating validated compositional imaging and biochemical markers into clinical pathways could enable earlier diagnosis, refined risk stratification, and biomarker-guided interventions to prevent irreversible cartilage loss. Supporting this view, Kraus et al. [15] demonstrated that serum biomarkers can predict incident knee OA up to eight years before radiographic changes, consistent with a long preclinical continuum influenced by genetic, environmental, and joint-specific aging factors. Nevertheless, the conventional “wait-and-see” or “retardist” approach—a form of clinical/therapeutic inertia, in which treatment is postponed until symptoms are evident—relies on outdated assumptions and neglects the robust evidence that early cartilage damage can be irreversible and precedes detectable clinical or radiographic manifestations. This temporal disconnect between early tissue pathology and established disease, exacerbated by persistent misconceptions, remains a major obstacle to the implementation of timely mechanism-based therapies. Abandoning the delayed-treatment approach will require rigorous analytic and clinical validation, including standardized assays and imaging protocols, prespecified prognostic thresholds, independent prospective calibration, and randomized or pragmatic trials that show improved patient-centered outcomes, cost-effectiveness, and acceptable safety. Addressing these steps is essential for early detection to translate into durable, disease-modifying care.

As mentioned above, a common misconception is that OA is an inevitable consequence of aging, whereas accumulating evidence demonstrates that disease onset is driven by early, modifiable molecular, biomechanical, and metabolic perturbations. This belief leads many individuals to overlook early signs and delay preventive action [21,63,64,95]. In reality, OA is a progressive, multifactorial condition, and its subtypes and clinical courses vary widely among patients [21,96,97]. Age- and environment-associated epigenetic modifications (DNA methylation, altered histone marks, dysregulated noncoding RNAs) likely contribute to interindividual discordance in outcomes, consistent with approximately 50% concordance in monozygotic twins, implying that progression is probabilistic rather than deterministic [98]. These distinctions have direct implications for pre-OA; operational definitions and early intervention strategies must account for baseline susceptibility, stratify by likely progression pheno-endotypes (e.g., rapid progressors versus stable/non-progressors), and prioritize cohorts and models that capture interacting drivers such as prior injury, poor alignment, abnormal mechanics, overweight/obesity, metabolic dysfunction, sex/hormonal status, and epigenetic background. Importantly, post-traumatic and idiopathic (non-traumatic) OA often follow different pathophysiological trajectories and may require distinct preventive and therapeutic approaches [21]. Rather than a single natural history, outcomes after joint insult range from a continuum from resilient individuals who remain stable to those who progress rapidly when synergistic drivers converge, underscoring the need for risk-stratified detection and phenotype-tailored intervention strategies.

Accordingly, OA should be viewed as “a common endpoint” rather than “a singular final pathway”. Although advanced disease converges on shared structural and clinical features, its development can arise from diverse etiological routes and mechanistic subtypes, such as post-traumatic, metabolic, postmenopausal, and age-associated phenotypes [69,99,100]. Clarifying these distinctions and addressing modifiable risks are essential for developing personalized therapies and effective public health strategies. The concept of pre-OA flows naturally from this heterogeneity: ultra-early, typically silent, structural and molecular changes mark a clinically relevant phase in which targeted prevention or interception may be possible. To translate this perspective into practice, priorities include standardizing acquisition and reporting, establishing prognostic thresholds (for example, composite imaging–biomarker–biomechanics scores calibrated against 3–5-year progression risk), and demonstrating that biomarker-directed early therapy improves long-term outcomes [14,15,26]. Operational steps should incorporate coordinated action: multistakeholder consensus on case definitions and minimal measurement sets; clinician and public education to counter misconceptions; pragmatic primary-care risk tools with stratified testing pathways; and pilot implementation studies that evaluate feasibility, clinical utility, cost-effectiveness, and effects on health equity. Pursuing these steps will enable risk-stratified detection and interventions tailored to distinct OA pathways.

## 5. Pre-OA: Defining the Window Before Structural Damage

Distinct from early OA, the pre-OA construct has gained traction as a potentially actionable pre-disease stage, but it is variably defined across studies, with heterogeneity in terminology, inclusion criteria, and measurement methods [18]. This lack of consensus hinders study comparability, complicates meta-analyses, and delays the development of evidence-based guidance for early intervention and implementation. In practice, definitional ambiguity impedes the systematic deployment of lifestyle programs, targeted exercise regimens, and evaluation of candidate chondroprotective therapies, and complicates policy decisions regarding coverage for preventive services. Establishing harmonized, consensus-driven definitions of pre-OA is therefore a priority. Such a definition should be multidimensional—explicitly specifying core domains (clinical features, compositional imaging sequences and thresholds, analytically validated molecular biomarkers, and biomechanical measures), a stepwise framework for pragmatic clinical application encompassing primary care risk scoring, intermediate testing, and advanced confirmation, and pre-specified validation criteria (analytic reproducibility, prospective prognostic performance, and external generalizability across diverse populations). Coordinated consensus (Delphi) processes that include clinicians, researchers, patients, regulators, and payers will accelerate translation and inform policy decisions, including potential insurance coverage for validated preventive interventions.

In the pre-OA stage, subtle perturbations in chondrocyte function disrupt ECM homeostasis within the hyaline cartilage, serving as a central driver of disease pathogenesis. These early changes, including increased expression of matrix-degrading enzymes, loss of proteoglycan synthesis, cellular pre-senescence, and altered mechanotransductive signaling, can compromise matrix homeostasis long before symptoms arise. In this context, it is useful to distinguish disease (the underlying biological and pathological alterations within the diarthrodial joint) from illness (the patient’s subjective experience of symptoms such as pain and functional limitation) [21,101,102]. Although pain is often the first symptom reported by OA patients, it commonly appears only after substantial subclinical pathology [95]. Clinical practice that prioritizes addressing illness after disease is already advanced, therefore missing opportunities for early, disease-modifying intervention. Operationalizing a pre-OA construct requires validated biomarkers and imaging metrics that capture chondrocyte dysfunction and ECM compositional changes (for example, compositional MRI, targeted molecular assays, or biomechanical monitoring), together with prospective evidence that these attractor-based markers predict progression and are modifiable by timely intervention. Clear communication with patients about the distinction between “disease” and “illness” and shared decision-making around screening and early intervention are essential as detection strategies are implemented [21].

Over half of symptomatic knee OA cases occur in people under 65, many of whom face decades of disability [103]. Despite this substantial burden, research on pre-OA remains limited, particularly for idiopathic cases, which comprises the majority of clinically diagnosed OA. Mechanistic and translational studies have disproportionately emphasized post-traumatic OA (PTOA), which accounts for only ~12% of OA cases, producing an uneven evidence base that leaves idiopathic pathways poorly characterized [104]. This imbalance is important because idiopathic pre-OA likely follows distinct etiological and temporal trajectories from PTOA and therefore requires different detection and prevention strategies. Identifying biomarkers, imaging signatures, biomechanical markers, and clinical risk profiles linked to idiopathic pre-OA is essential for developing effective screening tools and targeted interventions. The seminal contributions of Chu and colleagues elegantly integrated experimental biomechanics with molecular pathobiology in PTOA [37,38,39,40,42,43,44], providing a rigorous framework that should be tested in analogous large-scale biomarker-guided longitudinal studies of idiopathic OA. Priority actions include assembling diverse prospective cohorts with repeated compositional MRI, molecular profiling, and wearable biomechanics; deriving and externally validating composite prognostic scores to identify high-risk (rapid-progressor) subgroups; and conducting biomarker-guided prevention trials that assess clinical and structural endpoints, safety, cost-effectiveness, and equity. Addressing this gap will expand preventive options for younger at-risk populations and improve the long-term outcomes.

Despite growing attention to early-stage OA, inconsistent terminology and limited explicit characterization of the preclinical phase hinder comprehensive literature integration and may obscure key knowledge gaps. A targeted PubMed query illustrates a pronounced imbalance in the literature: terms explicitly indexing the pre-OA concept are rare relative to the far larger body of work on early OA. Using the search terms “Pre-Osteoarthritis” [All Fields] and “Pre-Osteoarthritis” [Title] (search conducted on 15 August 2025) returned 39 and 8 records, respectively, whereas “Early Osteoarthritis” [All Fields] and “Early Osteoarthritis” [Title] returned 1046 and 287 records, respectively. These descriptive counts, subject to the usual limitations of keyword choice, MeSH indexing, and database coverage, suggest that pre-OA as an explicitly labeled construct has been relatively underexplored. Historically, OA research emphasized early symptomatic or structural disease from the mid-1980s onward, although pioneering biochemical studies by Mankin and colleagues first suggested preclinical cartilage changes decades earlier [105,106]. Much contemporary pre-OA research has concentrated on PTOA cohorts [8,18,20,37,38,39,40,41,42,43,44,107], leaving idiopathic pre-OA, which accounts for the majority of clinically diagnosed OA, relatively neglected. To address this gap, I recommend a formal scoping review and bibliometric analysis, harmonization of keywords/MeSH terms, and research investments in large, longitudinal idiopathic cohorts with deep phenotyping to enable early intervention trials. These steps will clarify the evidence landscape and guide the strategic prioritization of pre-OA research.

Addressing OA at the pre-OA stage presents an important opportunity to prevent irreversible joint damage. Because threats to joint health arise early in life, prevention strategies should span the life course—beginning in childhood and continuing through adulthood—to reduce cumulative exposure, preserve matrix resilience, and delay progression to clinical disease [21]. Pre-OA after discrete joint injury (post-traumatic) often shows clearer biological and biomechanical signatures [38,40,42,44,107], but idiopathic pre-OA develops more gradually and lacks a single triggering event, making scalable early detection more challenging. Although high-resolution imaging and multimarker panels can detect early changes in post-traumatic cohorts, their cost, complexity, and limited availability reduce their feasibility for population screening in asymptomatic individuals. Until validated, accessible diagnostics exist for idiopathic pre-OA, a pragmatic dual strategy is advisable: (1) population-level prevention to reduce modifiable risks (weight management, physical activity, injury prevention, and metabolic control) and (2) targeted identification and intervention for individuals at higher predicted risk using pragmatic clinical risk scores and stratified diagnostic pathways. Translating this approach requires analytic and prognostic validation of candidate protein/miRNA/metabolite markers, prospective calibration of risk thresholds, economic evaluation, and pilot implementation studies that assess feasibility and acceptability. Drawing on community-based prevention models (e.g., oral health programs), OA prevention should aim for scalable and sustainable interventions that combine population measures with focused, evidence-based actions for those at greatest risk [21]. Just as routine oral hygiene shifted dental disease from inevitability to preventability, routine, mechanism-guided interventions in pre-OA could render OA the exception rather than the rule—preserving function and avoiding invasive care at the population level.

**Figure 3:**
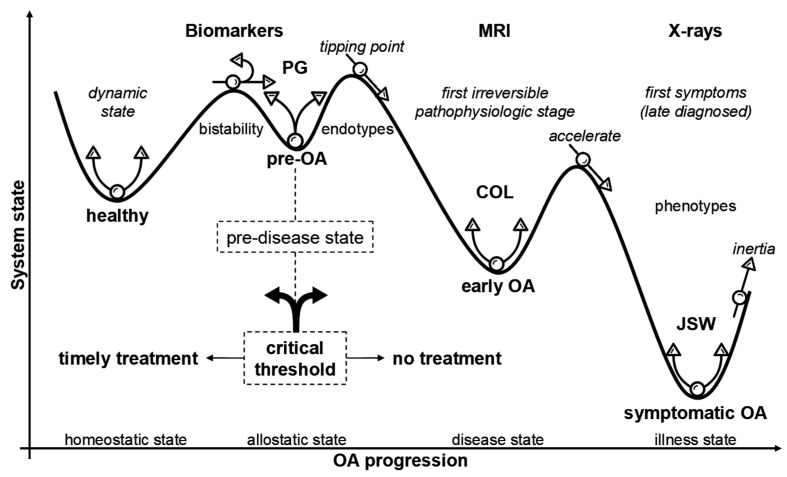
Linking the OA quasi-potential landscape to proteoglycan-link protein biology and candidate pre-OA signals. The schematic depicts the OA continuum—homeostasis → pre-deterioration/pre-OA → incipient/early OA → symptomatic/advanced OA—as a qualitative quasi-potential landscape of the cartilage–joint organ in which basins (attractors) and saddles (barriers) convey system resilience, metastability and bifurcation-type transitions. Large aggrecan proteoglycans form high-molecular-weight aggregates with hyaluronan, stabilized by hyaluronan–proteoglycan link proteins (e.g., the HAPLN family); reduction or loss of link proteins destabilizes these aggregates, increasing susceptibility to mechanical, enzymatic, or diffusive proteoglycan loss. Proteoglycan depletion may also occur independently through impaired synthesis (chondrocyte metabolic shifts), increased proteolysis (ADAMTS, MMPs), or inflammatory/oxidative stress. Notably, basins can represent long-lasting, non-progressive attractor states in which individuals remain clinically silent for years (slow or non-progressors), or they can reflect predisposed states that, under synergistic drivers, transition to rapid progression. This schematic underscores a paradigmatic conceptual shift away from reliance on descriptive, late-stage biomarkers used for phenotype identification toward the adoption of predictive, mechanistic biomarkers that enable early endotype delineation and actionable pre-disease detection [4,14,27]. Candidate diagnostic signals map onto dynamical regimes: pre-OA—subtle compositional MRI abnormalities and modest shifts in link-protein levels (HAPLN1/HAPLN2) and early matrix fragments (COMP, aggrecan neo-epitopes); critical/transition—rising degradative fragments, increased variance in functional metrics, and emerging focal cartilage defects on MRI (this region warrants careful organ-level assessment including meniscus, synovium, subchondral bone, ligaments, periarticular muscle/fat and neurovascular inputs); early OA—persistent compositional defects and collagen-matrix disorganization with sustained biomarker elevations; symptomatic/advanced OA—overt structural degeneration such as reduced JSW on radiograph and BMLs accompanied by clinical symptoms. Abbreviations: pre-OA, pre-osteoarthritis; PG, proteoglycan; MRI, magnetic resonance imaging; JSW, joint space width; X-ray, radiograph.

A central challenge in OA research is defining the earliest inflection points and biological trajectories along the continuum from pre-OA to symptomatic disease and distinguishing reversible metabolic instability from irreversible structural decline. As noted above, pre-OA is increasingly recognized as a discrete, potentially reversible state characterized by subtle biochemical, mechanical, and metabolic perturbations in chondrocytes that precede overt matrix fibrillation and widespread joint involvement [18,40]. During this phase, the collagen network remains largely intact and extra-cartilaginous tissues minimally affected, delineating a pragmatic therapeutic window in which targeted interventions may feasibly restore joint stability. As emphasized by Chu et al. [38,40], a detailed understanding of these incipient cellular and biomechanical interactions is indispensable for the rational design of targeted interventions aimed at delaying or preventing disease progression. Figure 3 schematically illustrates how the molecular destabilization of proteoglycan–link complexes can shift the joint toward an alternative attractor state, reflecting a reduced capacity to maintain homeostasis. Within this conceptual landscape, pre-OA can be viewed as a shallow, metastable basin in which temporally overlapping and partially redundant catabolic, inflammatory, and metabolic pathways synergistically erode resilience, implying that successful prevention will require rational, biomarker-guided combination strategies capable of simultaneously modulating complementary molecular and biomechanical nodes. Importantly, not all deviations from the baseline represent pathological attractors, as physiological modifiers, such as puberty, pregnancy, or menopause, may induce transient adaptive tissue states that must be distinguished from genuine pre-disease trajectories. Once the system transitions into the deeper attractor corresponding to established OA—marked by cartilage remodeling, osteophyte formation, and subchondral bone alteration—the barriers to reversal increase substantially, and interventions are more likely to create a new, less severe symptomatic basin than to reinstate the original healthy state. These principles underscore the need for validated biomarker thresholds, harmonized multimodal imaging frameworks, and longitudinal sex- and age-stratified cohorts capable of discriminating adaptive variation from reversible pre-OA [23,35]. Such frameworks will be essential for determining actionable, mechanism-based triggers for ultra-early intervention and evaluating whether preventive strategies can sustainably reshape the joint-state landscape toward a more resilient attractor.

Although not all individuals with pre-OA progress to early OA, and among those who do, progression can be rapid, slow, or remain clinically silent, developing precise radiologic and biochemical biomarkers to predict these trajectories is essential for personalized intervention. Current diagnostic tools often fail to detect subtle pre-OA changes, leading to missed opportunities for timely intervention [38]. Accurate predictors should meet clear criteria: analytic reproducibility, prospective prognostic performance over pre-specified time horizons, and demonstrable change with effective interventions. As OA advances, early disease is frequently accompanied by subclinical synovitis, an inflammatory milieu in the synovial membrane characterized by elevated mediators (e.g., prostaglandins and nitric oxide) that contribute to pain and swelling but are often attributed to aging, delaying diagnosis [92,108]. Inflammation-driven ECM remodeling disrupts chondrocyte homeostasis and diminishes regenerative capacity [109], and progressive loss of collagen network integrity indicates irreversible structural failure with downstream synovial and subchondral bone pathology [110,111,112,113]. Ultimately, persistent low-grade inflammation and chondrocyte terminal differentiation can promote endochondral ossification, subchondral sclerosis, and joint collapse, frequently necessitating invasive interventions such as joint replacement (see Figure 2). These mechanistic insights underline the imperative for early detection, harmonized biomarker pipelines, and intervention trials designed to preserve cartilage integrity and test whether interception can alter the natural history of OA.

Furthermore, the transition from pre-OA to early OA involves a complex interplay of mechanical, biochemical, and cellular processes that disrupt cartilage homeostasis. Initial loading insults induce subtle biochemical changes and the release of matrix-derived degradation products (MDPs) and inflammatory mediators, which together compromise matrix integrity [108,114,115]. The early loss of proteoglycans and incipient collagen fibrillation increase the tissue water content and hydraulic permeability. Mechanically, this elevated permeability diminishes interstitial fluid pressurization during loading and reduces the dynamic stiffness. Reduced fluid support forces greater tensile and shear strains on the collagen network, concentrating mechanical stress and accelerating fibrillar disruption. Early inflammatory signaling, marked by elevated cytokines such as interleukin-1β (IL-1β), interleukin-6 (IL-6), and tumor necrosis factor-α (TNF-α), and increased prostaglandin and nitric oxide production, drives the upregulation of catabolic enzymes, including aggrecanases (ADAMTS family) and matrix metalloproteinases (MMPs), accelerating aggrecan and collagen breakdown. Accumulation of degradation products in the synovial fluid amplifies local inflammation, creating a self-reinforcing loop that accelerates structural deterioration. Concurrently, early changes in synovial fluid composition, such as reduced HA and PRG4 levels and altered electrolyte balance, impair lubrication and exacerbate mechanical wear [116]. These processes have important translational implications. Although some early interventions can slow OA progression, loss of collagen network integrity marks a turning point that is difficult to reverse completely [110,111,117,118,119]. Collagen has an exceptionally slow turnover rate, with a half-life exceeding 100 years, rendering the accumulated damage permanent [73,120]. Chu et al. [42] observed that even in cases where articular cartilage appeared arthroscopically normal, early abnormalities such as cartilage softening—an indicator of underlying collagen network damage—were frequently present, while the articular surfaces remained intact. These findings highlight the critical importance of detecting pre-OA, as it offers a valuable window for timely intervention to potentially halt or delay the disease progression.

Focusing on pre-OA rather than waiting for early OA is essential for prevention because initial symptoms (mild discomfort, stiffness) are often subtle and misattributed to aging, and public and clinical awareness of early disease is limited [66,121,122]. Consequently, early signs may be overlooked, and opportunities to modify risk factors or deploy preventive measures are missed. Several molecular candidates have emerged as promising early indicators. Cross-linked C-telopeptide fragments of type II collagen (CTX-II) have been detected in synovial fluid, serum, and urine, and correlate with cartilage degradation [123]. Serum procollagen type IIA N-terminal propeptide (sPIIANP) and type-II collagen helical peptide (sHELIXII) reflect aspects of cartilage metabolism and merit further study as prognostic markers [124]. Elevated CTX-II levels show prognostic potential for stratifying patients at higher risk of progression, but prospective validation, harmonized assays, and defined predictive thresholds are required before routine clinical use. Complementary structural work by Tschaikowsky et al. indicates that regions that appear macroscopically intact can nonetheless exhibit nanoscale collagen fiber thinning and fibrocartilage-like remodeling, suggesting microstructural compromise early in the disease and identifying mean collagen fiber thickness as a potential research diagnostic target [119]. However, the measurement techniques for such nanostructural markers are currently research-grade and not yet scalable. Importantly, because collagen turnover is exceedingly slow, accumulated collagen damage is largely permanent; hence, reliance solely on collagen damage markers for screening would limit preventive opportunities. Moving forward requires validated, sensitive measures of ultra-early ECM and synovial change (compositional MRI, synovial fluid proteomics/metabolomics, targeted molecular assays, and emerging optical/spectroscopic approaches), prospective prognostic validation of multimodal biomarker panels that integrate serum/urine markers, imaging, and clinical risk, and randomized and implementation trials that test whether timely, mechanism-matched interventions (load modification, targeted anti-catabolic/anti-inflammatory therapies, metabolic modulation) can alter the trajectory toward irreversible structural disease.

## 6. Matrix Degradation-Associated Secretory Endotype: Unveiling Its Latent Effects on Normal Gait

**Figure 4:**
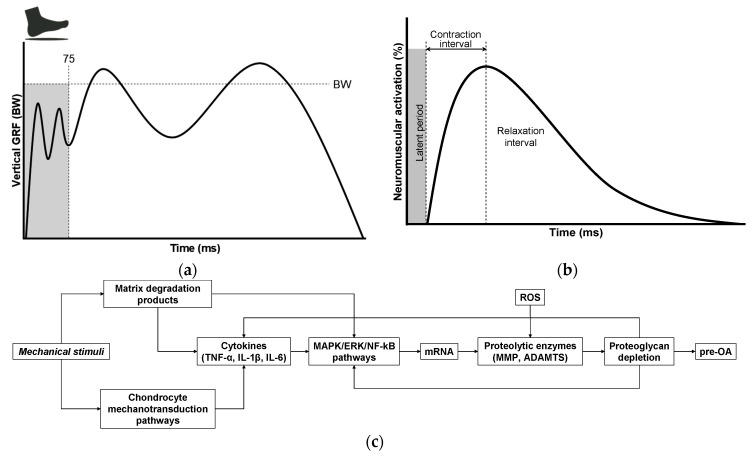
Mechanical triggers of cartilage degeneration leading to pre-OA. (**a**) HST during a typical gait cycle. The vGRF was plotted and normalized to %BW across the stride cycle. The shaded region highlights the HST (approximately 0–15% of the cycle; vertical dashed line = heel contact), where vGRF rapidly rises to its early peak. Initially, energy is absorbed (negative power), followed by a swift release (positive power) as the foot pushes off, thereby capturing the cyclic mechanical load that impacts the joint tissues. (**b**) Muscle response latency to gait cycle mechanical stimuli. Typical muscle tension (arbitrary units) over time (ms) following the gait stimulus. The phases shown were initial stimulus (heel contact at 0 ms), latency period/pre-activation delay, contraction (tension increase), and relaxation. Reflexive latencies commonly fall within 50–100 ms but vary with the muscle group and gait speed. (**c**) Mechanistic pathways linking mechanical loading to pre-OA. Mechanical stress (HST) triggers chondrocyte mechanotransduction and activates downstream signaling cascades (e.g., MAPK/ERK and NF-κB), increases intracellular ROS/RNS and pro-inflammatory cytokine (TNF-α, IL-1β, and IL-6) production, and upregulates proteolytic enzymes (MMPs and ADAMTS). The resultant degradation of aggrecan generates MDPs and DAMPs that further amplify inflammation and ECM proteolysis in a feed-forward loop, driving progressive cartilage deterioration and a matrix-degradation-associated secretory endotype. Adapted from: [87,128,129,131,132,135,144,145,146,147,148,149]. Abbreviations: ADAMTS, a disintegrin and metalloproteinase with thrombospondin motifs; BW, body weight; DAMPs, damage-associated molecular patterns; ERK, extracellular signal-regulated kinase; IL-1β, interleukin-1 beta; IL-6, interleukin-6; MAPK, mitogen-activated protein kinase; MDP, matrix-derived degradation products; MMP, matrix metalloproteinase; NF-κB, nuclear factor kappa B; ROS, reactive oxygen species; TNF-α, tumor necrosis factor-alpha.

OA is now recognized as a multifactorial disorder in which the mechanical environment of the joint plays a central permissive and provocative role [125,126,127]. Habitual walking—performed thousands of times per day—can generate mechanically meaningful cumulative loading, despite being widely regarded as a low-impact activity [117,126]. A key feature of normal gait is the heel-strike transient (HST), an abrupt, high-rate rise in the vertical ground reaction force (vGRF) that follows heel contact (Figure 4a) and can be quantified by the peak vGRF, loading rate, and impact impulse [87,128,129,130,131,132,133,134,135]. Repeated high-rate loading imposes rapid strain and fluid pressurization in articular tissues and can promote microdamage over many cycles. Importantly, HSTs occur across a broad population, including asymptomatic individuals [132], but their magnitude and rate are strongly modulated by body mass, gait speed, muscle recruitment patterns, alignment, and neuromuscular coordination [136,137,138,139,140,141,142,143], which are factors amenable to intervention (e.g., weight reduction, gait retraining, strength training, walking speed modification, and shoe/orthotic or insole cushioning). Several experimental studies have reported very large impulsive loads in poorly coordinated strikes, yet magnitudes vary significantly with measurement method and experimental context. For example, O’Connor et al. [87] reported that minor incoordination during heel strike can markedly impair leg deceleration and, under specific experimental conditions, produce impulsive loads reported as high as ~65× body weight, illustrating how neuromuscular control failures can greatly amplify HST. Together, these observations motivate the hypothesis that frequent high-rate HST exposures can contribute to cumulative cartilage matrix damage and help trigger a matrix degradation-associated secretory endotype that links mechanical loading to biochemical and cellular pathology in pre-OA.

Although HSTs have been characterized for decades, their contribution to cartilage homeostasis and degeneration remains underappreciated. Accelerometer measurements indicate that the heel-strike impact transient propagates rapidly up the limb, with reported propagation velocities on the order of 10^2^ m·s^−1^, and transient peaks in the lower leg in some experimental settings approaching 610 m·s^−1^, corresponding to transit times on the order of milliseconds (approximately 2 ms to the knee, 4 ms to the hip, and 8 ms to the skull) [150,151]. These findings underscore that the knee acts as a primary shock-attenuating joint during the early stance, absorbing and redistributing high-rate loads [134,152]. To limit mechanical insult, the body relies on an integrated hierarchy of protective mechanisms: passive damping from the plantar (heel) fat pad and fascial compartments, structural support provided by peri-articular tissues (joint capsule, ligaments, and synovium), inertial dissipation through visceral soft-tissue motion, and—critically—active force modulation (mechanical buffering) via coordinated lower-limb muscle activity, which dynamically adjusts loading in response to movement and impact. These specializations of obligate bipedalism (robust plantar fat pad, longitudinal foot arch, limb alignment, and neuromuscular control) attenuate impact transients but also concentrate repetitive loads on the knee and hip, which is a biomechanical trade-off relevant to joint degeneration. Lower-extremity muscles perform coordinated eccentric (negative) work at the heel strike to dissipate impulsive energy, modulate limb deceleration, and protect diarthrodial joints [153,154,155]. Because HST magnitude, rate, and load absorption are modulated by body mass, gait speed, muscle coordination and joint biomechanics, these variables represent plausible, modifiable contributors to cumulative cartilage loading and OA risk and warrant targeted mechanistic studies using synchronized accelerometry, electromyography (EMG), and compositional cartilage measurements.

Given the mechanistic importance of muscle control and neuromotor coordination, the precise contribution of the neuromuscular system to OA onset and progression remains incompletely defined. Effective shock absorption during gait depends on rapid activation of muscles, typically occurring within 50–100 ms following a mechanical stimulus [129,144,145,147]. This reflexive response is mediated by proprioceptive afferents with conduction velocities of approximately 30–120 m·s^−1^, enabling rapid muscle engagement to attenuate impact forces [147,156,157,158,159]. Short-latency muscle responses to unexpected perturbations during the swing phase highlightthe role of flexor–extensor co-contraction in controlling joint stiffness. This co-contraction serves as an immediate defensive mechanism, stabilizing the diarthrodial joint and preparing for longer-latency adjustments to variations in load or terrain [147]. As shown in Figure 4b, the brief pre-activation delay—termed the latent period—represents a critical vulnerability in which the joint is exposed to mechanical impact before muscle activation can effectively mitigate the force. Because this latency (commonly up to ~100 ms) can exceed the very short timescale over which the foot must be decelerated at the heel strike (5–20 ms), a temporal mismatch may allow excessive peak loading to reach the joint, potentially triggering cartilage microdamage and the earliest pre-OA changes. However, the precise pathobiological mechanisms linking the transmitted mechanical load to molecular cartilage degeneration remain poorly understood.

This model is testable. High-fidelity measurements—surface EMG synchronized to force plates or validated wearable force/pressure sensors, along with 3-D kinematics—can precisely quantify muscle onset latencies (including short-latency reflexes) relative to the transmitted HST magnitude. These biomechanical metrics can be prospectively related to early biochemical markers and compositional MRI changes. If a causal chain exists, interventions that enhance neuromuscular timing (e.g., perturbation training, task-specific gait re-education, progressive strengthening) or footwear/orthotic strategies that preserve or augment plantar afferent feedback should reduce transmitted loading and favorably alter biomarker trajectories. Conversely, interventions that primarily attenuate plantar cutaneous feedback, such as very thickly cushioned shoes, extreme off-loading orthoses, or overly compliant insoles, may lower measured peak HST but diminish plantar afferent signalling, alter short-latency reflex excitability, and in some studies, produce delayed or modified EMG onsets and compensatory gait adaptations [140,141,142,143]. Thus, devices that purely diminish plantar sensation could paradoxically prolong the effective pre-activation delay and merit careful evaluation in mechanistic and longitudinal studies.

Despite significant advances in OA research, critical aspects of its pathogenesis remain poorly understood, particularly key early triggers that convert mechanical perturbation into biochemical pathology. Emerging evidence supports a mechanistic cascade in which mechanical loading, particularly repetitive high-rate impacts, disrupts the ECM and liberates MDPs that function as damage-associated molecular patterns (DAMPs). As illustrated in Figure 4c, these fragments engage chondrocyte mechanotransduction and innate immune pathways (for example, via MAPK/ERK and NF-κB signaling), promote the synthesis of pro-inflammatory cytokines (TNF-α, IL-1β), and upregulate genes encoding proteolytic enzymes (MMPs, ADAMTS) [91,148,149,160,161,162,163,164,165,166,167,168,169]. The resulting proteoglycan loss degrades cartilage biomechanics and sustains cytokine production, establishing a self-reinforcing cycle of matrix degradation and inflammation that accelerates the progression from pre-OA to established disease. This model identifies measurable nodes for early detection (matrix fragments, enzyme activity, phosphorylated signaling effectors) and tailored intervention (load reduction, pattern recognition receptor (PRR)/modulator blockade, selective protease inhibition) and calls for coordinated in vitro loading studies, controlled animal impact models, and longitudinal human cohorts that link gait-derived loading metrics to early molecular and compositional imaging changes.

## 7. Load-Induced Release of ECM Fragments: Mechanisms, Signaling, and Biomarker Potential

**Figure 5:**
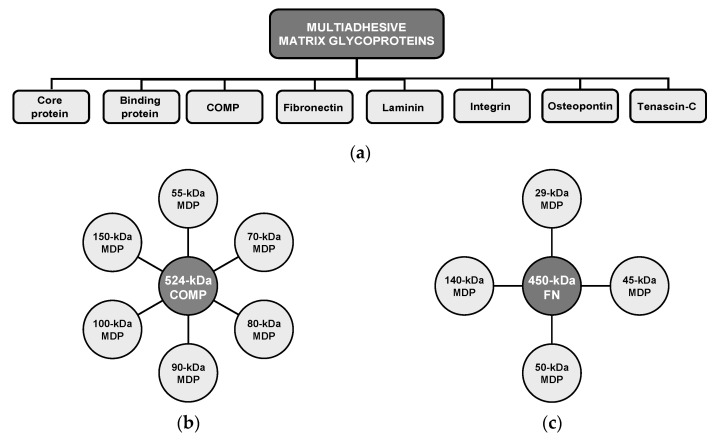
Schematic overview of principal cartilage matrix glycoproteins and their major MDPs implicated in downstream catabolic signalling. (**a**) Multi-adhesive glycoproteins in the articular cartilage. Adhesion glycoproteins, particularly COMP and fibronectin, are abundant ECM constituents that, together with water, proteoglycans, and collagens, maintain matrix cohesion, Gibbs–Donnan swelling pressure, and load-bearing function. This panel lists the principal glycoproteins and summarizes their known roles in structural support, cell adhesion, and mechanoadaptive signaling. (**b**) COMP degradation and major MDPs. A circular schematic depicts the major COMP-derived fragments arranged clockwise by increasing molecular weight, with annotated approximate masses (kDa). (**c**) A parallel circular schematic showing the principal FN-fs, ordered by increasing molecular weight, with annotated fragment sizes (kDa).

Human weight-bearing joints endure hundreds of millions of loading cycles over a lifetime, yet articular cartilage normally preserves functionality through the synergistic actions of water, proteoglycans, collagen, and adhesion glycoproteins—notably cartilage oligomeric matrix protein (COMP) and fibronectin (FN)—which maintain ECM architecture and intra-tissue hydrostatic pressure (Figure 5a) [146,162,170,171,172,173,174,175,176,177,178,179,180,181,182]. Mechanical stress, inflammatory processes, and proteolytic activity disrupt this balance, releasing COMP and FN from the ECM and generating MDPs that accumulate in cartilage and synovial fluid, with markedly elevated levels in OA [162,170,183]. While certain fibronectin fragments (FN-fs) at low concentrations (e.g., 29-kDa MDP) can transiently stimulate proteoglycan synthesis and anabolic factor release, such as TGF-β and IGF-1 [170], persistent or high-level exposure induces catabolic signaling, potentiates ECM breakdown, and accelerates degeneration [160,161,162,178]. Acting as DAMPs, MDPs engage innate immune receptors (e.g., TLR2/TLR4, CD44) and activate MAPK/ERK and NF-κB pathways, sustaining cytokine production and protease expression in a self-amplifying loop [148,149,162,168,184,185,186]. In support of a causal chain from ECM fragmentation to inflammation and proteolysis (Figure 4c), Jung et al. [148] reported that the breakdown products of chondroitin sulfate induce hypertrophy-like changes in chondrocytes with increased MMP-13 and ADAMTS5 expression, oxidative stress, and NO production through TLR2/TLR4 signalling, effects that were ameliorated by pharmacologic TLR2/4 inhibition, thereby functionally linking ECM fragmentation to protease induction and suggesting PRR blockade as a potential interception strategy. These dynamics identify measurable nodes (COMP/FN fragments, aggrecan fragments, MMP/ADAMTS activity) and therapeutic targets (protease inhibition, PRR modulation, load reduction) for early detection and interception, but prospective validation in longitudinal human cohorts is required.

COMP contributes to cartilage structural integrity by binding key ECM components, including type II and type IX collagens, and by supporting collagen fibril assembly and matrix cohesion. This glycoprotein also functions as an anchoring component that facilitates interactions critical for chondrocyte regulation and cartilage metabolism. Fragmentation and release of COMP occur with matrix disruption under cyclic mechanical loading, and liberated COMP fragments can participate in inflammatory signaling that may exacerbate joint degeneration (Figure 5b). Importantly, serum COMP is mechanosensitive in physiologically normal individuals, and controlled exercise studies have reported transient increases after walking that return to baseline over hours [174,176,187,188,189,190]. For example, Mündermann et al. [174] reported that serum COMP levels in healthy adults were measured just before, 30 min after, and at 0.5, 1.5, 3.5, and 5.5 h following a 30-min walking exercise, with these results compared to a control group that remained sedentary during office work. An initial 9.7% increase in COMP was observed 30 min postexercise, returning to baseline by 3.5 h, followed by a second 7% increase at 5.5 h [174]. This early increase likely correlates with temporary cartilage surface wear, whereas the subsequent increase may reflect enhanced COMP synthesis aimed at joint surface repair [174]. Similarly, Andersson et al. [187] observed that serum COMP levels increased one hour after a one-hour treadmill walk at the pace chosen by each participant, with levels returning to baseline two hours post-exercise. These kinetics likely reflect increased matrix turnover and short-term release of COMP rather than inevitable tissue loss. Therefore, the translational use of COMP requires standardized sampling (timing relative to activity and time of day), analytically validated assays, prospective correlation with compositional imaging and clinical outcomes, and evaluation of COMP within multimarker panels to distinguish physiological loading responses from early pathologic signals.

FN is a high-molecular-weight ECM glycoprotein that binds cell-surface integrins (for example, α5β1), interacts with collagen and proteoglycans, and also supports matrix assembly, repair, and chondrocyte adhesion. As shown in Figure 5c, proteolytic cleavage of native FN, driven by mechanical stress and proteases, including MMPs and ADAMTS, generates bioactive FN-fs that modulate chondrocyte behavior [178,191]. Certain FN-fs (e.g., ~29-kDa species) have been reported to transiently stimulate anabolic signals at low concentrations, but persistent or high-level accumulation potently induces pro-inflammatory cytokines and upregulates matrix-degrading enzymes, thereby amplifying ECM breakdown. FN-fs signal via integrins and innate receptors (e.g., TLR2/TLR4) to activate the MAPK/NF-κB pathways, positioning them as both biomarkers and mediators of the switch from physiological turnover to pathological degradation. Accordingly, these ECM fragments are candidate mechanistic mediators and biomarkers of the pre-OA transition but require fragment-specific assays and prospective validation. To move from mechanism to clinic, precise identification of FN-f species (mass and cleavage site), robust assays (targeted ELISA or liquid chromatography, and/or mass spectrometry), and prospective correlation with imaging and clinical outcomes are required [4,14,15,26,80].

## 8. Breaking the Balance: Ultra-Early Cartilage Dysregulation in Pre-OA

Pre-OA denotes a putative subclinical stage between joint health and early OA, in which subtle biochemical, cellular, and biomechanical perturbations precede detectable ultrastructural damage and clinical symptoms. These early changes—often reversible—typically involve initial loss or redistribution of proteoglycans, incipient shifts in matrix turnover and low-grade inflammatory signalling while the collagen network remains structurally preserved [11,12,13,70,192,193,194]. Because conventional imaging and routine clinical assessment frequently miss these changes, advances in quantitative imaging—compositional MRI (T2/T1ρ/dGEMRIC), Raman and other spectroscopies, sensitive serum/urine biomarkers (CTX-II, COMP, sPIIANP), and gait/HST metrics—are critical for identifying at-risk individuals and defining actionable windows for intervention [4,17,23,47]. Inter-joint variability (e.g., knee versus ankle) in loading, structure, and repair capacity further complicates operationalization; some diarthrodial joints follow rapid post-insult trajectories, while others remain stable for years [195,196]. Determining whether pre-OA should be treated as a discrete clinical entity or as a dynamic point on a degenerative continuum has major implications for screening thresholds, trial design, and prevention strategies aimed at restoring matrix homeostasis before irreversible collagen loss.

**Figure 6:**
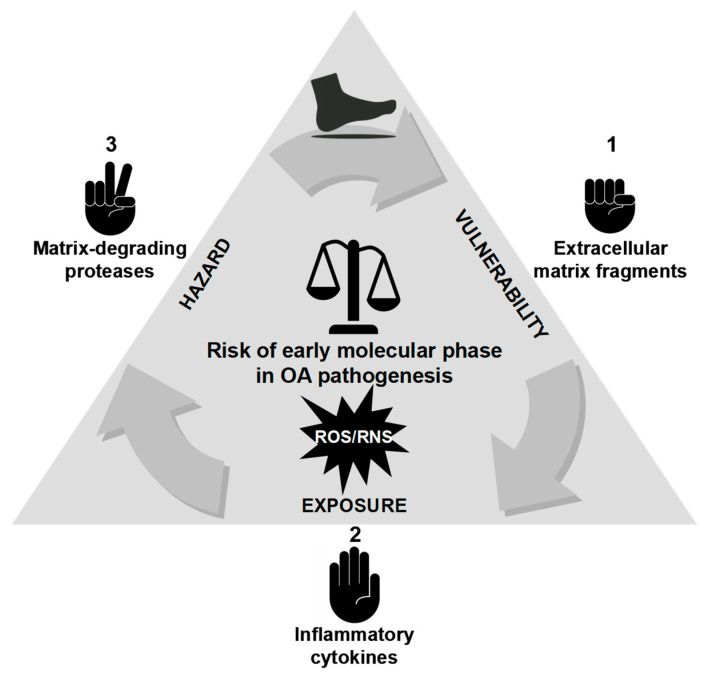
Conceptual model of OA development centered on the MDP–cytokine–protease triad, illustrated using the “rock–paper–scissors” analogy and their self-amplifying interactions. Mechanical loading or early matrix disruption releases ECM fragments (the “rock”), which act as DAMPs to trigger pro-inflammatory cytokine signaling (the “paper”) and activate intracellular pathways (e.g., MAPK/ERK, NF-κB). These signals upregulate proteolytic enzymes (the “scissors”), driving aggrecan and collagen degradation and generating further matrix-derived fragments. The resulting self-amplifying cycle of mechanically driven inflammation (termed “mechaflammation”) and matrix loss perpetuates early cartilage dysregulation and OA progression. Oxidative stress, driven by ROS and RNS such as superoxide, hydrogen peroxide, and nitric oxide, amplifies the inflammatory–catabolic loop. In early-stage OA, these species potentiate cytokine signaling (IL-1β, TNF-α), enhance the activation of matrix-degrading proteases (MMPs, ADAMTS), and further promote chondrocyte stress and matrix breakdown. Targeted interventions that interrupt one or more nodes of this loop, such as load modification, PRR/TLR antagonism, selective protease inhibition, or antioxidant strategies, represent candidate disease-modifying approaches.

To elucidate pre-OA pathogenesis, the dynamic interplay among ECM fragments, pro-inflammatory cytokines, and proteolytic enzymes can be conceptualized as a self-reinforcing feedback loop that drives early stage cartilage degradation (Figure 6; Table 2). In this model, ECM fragments (for example, aggrecan-derived peptides, COMP, and FN-fs) released by mechanical stress or oxidative damage act as initial triggers and function as DAMPs that signal via pattern recognition and adhesion receptors (e.g., TLR2/4, CD44) to the surrounding joint milieu [148,149,162,175,182,197]. These fragments stimulate chondrocytes and synovial cells to produce pro-inflammatory cytokines (IL-1β, IL-6, and TNF-α), which amplify inflammation and induce protease expression [108]. Upregulated proteolytic enzymes (MMPs and ADAMTS) catalyze aggrecan and collagen degradation, generating further ECM fragments and perpetuating the cycle (Figure 6). Oxidative stress (increased ROS and RNS activity) exacerbates these processes by enhancing cytokine production and protease activation [198,199,200,201,202,203,204,205]. Crucially, this cascade can manifest in the absence of clinical symptoms or structural damage, offering a potential window for intervention. Model validation requires integrated studies that combine high-fidelity loading metrics, serial molecular sampling, and compositional imaging to define temporal relationships, prognostic thresholds, and effective interception points.

Overall, the mechanoinflammatory model frames pre-OA as an early, self-sustaining disturbance of matrix homeostasis, wherein mechanical loading, ECM fragmentation, low-grade inflammation, and proteolysis interact to precipitate structural failure. Central to this concept is the ultra-early loss of the cartilage proteoglycan aggrecan—particularly within the narrow pericellular matrix immediately surrounding chondrocytes (the chondron)—which compromises osmotic swelling pressure and compressive stiffness and thereby reduces tissue resistance to mechanical stress [206]. Aggrecanolysis, most commonly mediated by ADAMTS-4/5, typically precedes collagen network breakdown and functionally links molecular degeneration to later structural failure. In addition to primary catabolic mediators (e.g., COMP and FN-fs) released during physiological loading, aggrecan-derived proteolytic fragments (e.g., ARGS neoepitopes) constitute secondary mediators of matrix catabolism and therefore represent promising early molecular biomarkers that warrant assay standardization and prospective validation using compositional MRI as a reference [4,26,27,99]. Because ARGS species reflect nascent proteoglycan loss, they have the potential to offer temporal sensitivity for detecting the earliest catabolic events, making them attractive both as diagnostic markers of pre-OA and as pharmacodynamic readouts for aggrecanase-targeted therapies. Realizing this potential will require (i) rigorous assay harmonization and cross-platform validation, (ii) characterization of compartmental dynamics and clearance (synovial fluid versus serum/plasma/urine), (iii) demonstration of specificity for aggrecanase cleavage versus other proteolytic processes, and (iv) prospective correlation of biomarker trajectories with compositional MRI changes (e.g., T1ρ/T2) and clinically meaningful outcomes in well-phenotyped longitudinal cohorts. Complementing molecular readouts, single-cell transcriptomic studies have identified discrete chondrocyte subpopulations and trajectory states that anticipate hypertrophic differentiation and correlate with progression, implying definable intermediate cellular endotypes amenable to targeted intervention [207]. Intriguingly, emerging data indicate that intrinsic cartilage circadian clocks temporally regulate anabolic versus catabolic programs, raising the possibility that circadian disruption, together with early aggrecan loss, may act as proximal molecular triggers for pre-OA development. Testing these links will require integrated studies combining compositional imaging, fragment-specific assays, single-cell and spatial profiling, and controlled perturbation experiments.

## 9. Mapping Temporospatial Patterns of Pre-OA Onset and Its Transition to Early OA

### 9.1. Circadian Oscillations of Chondrocyte Clock Genes and Reciprocal Regulation of Matrix Turnover

Circadian rhythms—endogenous ~24-h cycles that organize physiology—are governed by an interplay between a central “master clock” in the brain and autonomous oscillators embedded throughout peripheral tissues [208]. The central pacemaker in the suprachiasmatic nucleus (SCN) synchronizes tissue-specific circadian rhythms via environmental light cues, ensuring coherent temporal regulation throughout the body. This hierarchical organization enables the temporal coordination of diverse tissue functions and highlights the importance of local clocks in maintaining tissue homeostasis. Cartilage exhibits robust local oscillators: proteomic profiling in mice has identified nearly 150 proteins with day–night abundance cycles, and transcriptomic studies have reported several hundred rhythmic mRNAs, including core clock components (BMAL1/ARNTL, CLOCK, PERs, CRYs) and many matrix regulators [209,210,211,212,213,214,215,216,217,218,219,220,221]. As summarized in Table 3, these rhythmic programs encompass both degradative enzymes (for example, MMP14, ADAMTS4) and their endogenous inhibitors (for example, TIMP4), implying that net matrix turnover is temporally gated by the coordinated expression of opposing pathways [213]. For example, BMAL1 directs the rhythmic expression of both anabolic and catabolic genes. Cartilage-specific loss of BMAL1 abolishes local clocks and accelerates OA-like changes in mice, indicating the functional relevance of clock control for tissue homeostasis. Persistent circadian disruption or chronic mechanical stress can induce epigenetic reprogramming, which may lock in the depressed expression of chondroprotective genes (for example, ACAN, COL2A1, SOX9) [222]. These stable molecular markers may convert transient rhythmic disturbances into a “memory” state that predisposes cartilage to degeneration. Complementing tissue-level circadian data, these observations have clinical relevance, as large-scale genomic analyses have linked many OA risk loci and effector genes (Table 3). A recent large-scale translational human genomics study of approximately 1.96 million individuals identified 962 independent associations and implicated approximately 700 effector genes, underscoring the genetic complexity of OA [22]. Clock gene changes may contribute to the disease phenotype by disrupting clock-controlled pathways, but they may also reflect the consequences of tissue damage. Importantly, many rhythmic transcript and protein changes are most pronounced early in degeneration rather than after the disease phenotype is fully established [209], suggesting that disruption of daily proteome timing may be a proximal contributor to OA pathogenesis rather than merely a late consequence.

Clinical OA symptoms often show diurnal variation—pain and stiffness fluctuate across the day—and daily cycles of activity and rest likely modulate disease biology by altering mechanotransduction and patterns of joint loading. Cartilage matrix turnover is strongly time-of-day dependent. In humans, several biomarkers of cartilage metabolism (e.g., serum COMP and PIIANP, urinary CTX-II, hyaluronan, KS-5D4, TGF-β1, and HELIX-II) exhibit peak values in the early day (early morning) after overnight rest and/or morning activity [124,174,219]. Core clock genes (BMAL1/ARNTL, PER1/2, CRY1, NR1D1/2) coordinate these rhythms across musculoskeletal tissues [210], and time-series transcriptomics have demonstrated that the cartilage clock temporally segregates ECM synthesis and degradation programs over the 24-h cycle [217]. Together, behavioral rhythms, local clocks, and mechanoresponsive signaling plausibly link daily activity patterns to ultra-early pre-OA joint changes. Because recent activity and sleep also influence biomarker levels, rigorous studies must standardize sampling time and record sleep/activity covariates to distinguish endogenous circadian effects from behaviorally driven variations.

Of particular relevance to the pre-OA concept, chondrocyte-specific Bmal1 knockout (cKO) mice develop focal lesions confined to the articular cartilage with no early detectable changes in the subchondral bone, synovium, or ligaments [224]. This cartilage-restricted phenotype contrasts with the whole-joint pathology of established OA and indicates that chondrocyte-autonomous circadian disruption is sufficient to initiate focal cartilage degeneration in this animal model. To strengthen causal inference, it is important to note whether deletion was constitutive or induced in adulthood, and complementary studies (inducible adult-onset KOs, chondrocyte-specific rescue, or controlled mechanical challenge) help distinguish between developmental and adult-onset effects. Nonetheless, the Bmal1-cKO model provides compelling experimental evidence that early cartilage-specific molecular dysregulation can precede joint-wide involvement and is consistent with the emerging operational definition of pre-OA.

BMAL1-dependent circadian rhythms are critical for cartilage homeostasis, and BMAL1 dysregulation perturbs anabolic–catabolic balance in chondrocytes. In BMAL1-deficient chondrocytes, the transcriptome shifts toward a catabolic program, characterized by reduced TGF-β signaling and altered NFAT activity, downregulation of chondroprotective factors (e.g., SOX9, ACAN, COL2A1), and induction of matrix-degrading proteases (MMPs, ADAMTS) [213]. This signaling imbalance provides a plausible molecular mechanism for the cartilage-restricted lesions observed in chondrocyte-specific Bmal1-cKO mice [224], indicating that chondrocyte-autonomous loss of BMAL1 can impair matrix anabolism and increase susceptibility to catabolic stimuli during the pre-OA phase before whole-joint involvement. Testing causality and therapeutic potential will require inducible adult-onset models, functional restoration experiments, and chrono-targeted interventions to determine whether restoring clock function or temporally aligning anabolic signaling can prevent disease progression.

Human data corroborate and extend murine findings. In osteoarthritic cartilage, BMAL1 expression is reduced, often accompanied by reciprocal changes in PER2, and experimental BMAL1 knockdown in otherwise healthy human chondrocytes induces proliferative and catabolic phenotypes—including upregulation of MMP13—that recapitulate key features of the OA chondrocyte program [211]. Yang et al. [225] demonstrated that BMAL1 functionally interacts with SIRT1 and that reduced BMAL1 in OA cartilage is associated with disturbed NAD^+^ homeostasis and amplified catabolic/inflammatory responses. Similarly, Akagi and colleagues [214] reported the suppression of BMAL1 and NR1D1 in OA tissue and showed that silencing these clock components perturbs TGF-β signalling in cultured chondrocytes, directly implicating circadian disruption in impaired anabolic and repair pathways in human cartilage. Collectively, these human cell and tissue data, together with the chondrocyte-selective Bmal1 cKO model [224], support a model in which chondrocyte clock dysfunction marks an early, cartilage-confined stage of disease and identify the BMAL1–SIRT1–NAD^+^ axis as a tractable target for early intervention. However, caution is warranted because many human studies are cross-sectional and confounded by disease severity, age, and comorbidities. Therefore, inducible humanized models, longitudinal tissue sampling, and pilot chrono-intervention trials are necessary to establish causality and therapeutic potentials.

BMAL1-dependent circadian regulation temporally orchestrates multiple transcriptional and post-translational signaling hubs. This coordination aligns the daily rhythmic balance between anabolic (matrix synthesis) and catabolic (degradative) programs in chondrocytes, with important implications for tissue homeostasis and disease susceptibility. Loss of BMAL1 in chondrocytes suppresses the anabolic TGF-β/SMAD2/3 axis while biasing signaling toward SMAD1/5 outputs and is associated with reduced expression of anabolic effectors (for example, SOX9, COL2A1, and ACAN) and increased production of matrix-degrading proteases, a constellation consistent with a net catabolic shift [213]. Mechanistically, NFATC2 is transcriptionally regulated by CLOCK and displays time-dependent expression in cartilage, providing a direct link between the peripheral clock and chondrocyte anabolic programmes [213]. Human cartilage data complement these findings: BMAL1 and NR1D1 are decreased in OA tissue, and RNAi of these clock genes in cultured human chondrocytes dysregulates networks that include TGF-β signaling and upregulates catabolic mediators (for example, MMP13) [211,214]. Pro-inflammatory signaling provides a bidirectional interface: IL-1β markedly suppresses rhythmic Cry1 and Per2::Luc oscillations in mouse cartilage via NF-κB–mediated interference with the Clock:Bmal1 complex, demonstrating that chronic NF-κB activation can effectively “shut down” the local clock and link inflammation to circadian dysfunction [223]. Likewise, BMAL1 loss elevates ERK phosphorylation and IL-6 production in mandibular chondrocytes—effects reversible by BMAL1 restoration or ERK inhibition—further linking circadian dysfunction to OA-like catabolic responses [213]. Thus, cartilage clocks intersect with the TGF-β, NF-κB, and MAPK pathways to maintain ECM homeostasis, and circadian mistiming biases signalling toward net degradation. These data should be interpreted cautiously because studies are often tissue- and species-specific and do not uniformly generalize across all diarthrodial joints. For example, despite substantial Bmal1 loss, murine hip cartilage appeared largely unchanged up to 6 months of age, whereas the knee cartilage is critically dependent on chondrocyte BMAL1 for integrity [213]. Such contrasting phenotypes likely reflect site-specific mechanical loading differences—an important entraining cue for cartilage circadian clocks—and may underlie regional vulnerability that promotes hyperosmolarity-driven rhythmic gene expression in chondrocytes [226].

Daily repetitive biomechanical loading is a primary determinant of cartilage physiology and functions as a time-setting (entraining) stimulus for circadian clocks. These clocks are mechanosensitive and respond to the matrix stiffness, viscoelastic properties, and related mechanical cues of their extracellular environment [227,228]. Diurnal loading–unloading cycles induce predictable fluctuations in cartilage osmolarity that act as potent synchronizers (“zeitgebers”) for intrinsic chondrocyte clocks, helping to preserve temporal order within the tissue [226,229]. Appropriately timed joint-loading exercise aligned with the human activity phase may therefore reinforce clock entrainment, whereas reinstating physiological osmotic cycles through intermittent controlled loading may have rehabilitative potential for matrix homeostasis. At the molecular scale, time-resolved proteomics demonstrates temporal clustering of functional pathways—translation, cytoskeletal remodelling, and glucose metabolism—peaking at defined times of day, a partitioning that likely optimizes simultaneous load-bearing and repair tasks [217]. Emerging evidence implicates circadian control of organelle homeostasis (autophagy, mitophagy, and mitochondrial function) as a determinant of chondrocyte resilience during the early stages of degeneration [230]. Together, these data indicate that circadian timing integrates extracellular mechanical cues, intracellular metabolic programs, and organelle health to preserve cartilage function, and that defining temporal windows of susceptibility will be critical for designing effective chrono-targeted interventions to delay or modify the transition from pre-OA to early OA. Further research is required to clarify the precise pathobiological mechanisms involved.

### 9.2. Metabolic Heterogeneity in Cartilage: Implications of the Thick Phenotype for Early Catabolic Onset

Although impaired nutrient supply to the articular cartilage has long been recognized as a potential source of metabolic stress, its role in disease initiation remains unclear. Cartilage critically depends on solute transport to sustain chondrocyte metabolism and subtle diffusion constraints can produce steep metabolite gradients across cartilage depth. Because cartilage is avascular, chondrocytes rely heavily on the diffusive supply of oxygen and glucose from synovial fluid for ATP production and matrix synthesis [231,232,233,234,235]. Even modest reductions in solute transport can progressively reduce matrix turnover and energy availability, ultimately inducing localized bioenergetic stress within the tissue [236,237]. A recent mathematical model of chondrocyte energy metabolism [235] showed that perturbations in the balance between glycolysis and oxidative phosphorylation (OXPHOS) can produce a bistable transition to a low-ATP pathological state. The model further predicts that under chronic hypoxia or sustained reductions in solute exchange, this pathological attractor is favored, producing persistent ATP deficits and progressive ECM loss, whereas physiological fluctuations in oxygenation associated with normal loading tend to preserve metabolic flexibility [235]. These model predictions provide a mechanistic rationale for how impaired solute exchange and prolonged low-motion states can drive persistent metabolic dysfunction and matrix loss in vivo. By linking depth-dependent diffusion deficits to a switch-like collapse of ATP synthesis, these results provide a mechanistic basis for the proposed “stagnant cartilage syndrome” (SCS) as a testable hypothesis that mechanistically associates decreased habitual joint motion and impaired synovial mixing amplifies thickness-driven solute limitations in anatomically thick cartilages (e.g., knee and hip), creating focal zones of oxygen and glucose deprivation, reduced glycolytic flux, and acute ATP depletion [195,196]. Energetic failure compromises PAPS-dependent glycosaminoglycan sulfation and aggrecan core protein synthesis, resulting in under-sulfated dysfunctional proteoglycans that reduce fixed-charge density and osmotic swelling pressure [232,238]. When mitochondrial compensation is overwhelmed, excess mitochondrial ROS (e.g., superoxide, hydrogen peroxide) and impaired mitophagy release mitochondrial DAMPs and potentiate NF-κB/MAPK and inflammasome signaling, upregulating ADAMTS and MMP activity, while impairing TIMPs. This proteolytic cascade accelerates aggrecan cleavage, further diminishing tissue hydration and interstitial fluid mobility, thereby establishing a localized self-reinforcing catabolic niche [239]. Thus, SCS emphasizes spatial metabolic heterogeneity, rather than a uniform decline in metabolism, as a plausible determinant of focal pre-OA onset. Empirical validation should combine matched explant diffusion assays, spatial ATP/metabolite imaging (e.g., biosensors or mass spectrometry imaging), real-time metabolic flux measurements, and the assessment of inflammasome activation, ROS/RNS production, and matrix-degrading enzyme activity, together with in vivo models that recapitulate reduced joint motion.

A reduction in the aggrecan-driven fixed-charge density alters the poroelastic behavior of cartilage and shifts the balance between fluid and solid load support. As interstitial water content and Donnan osmotic pressure fall, interstitial fluid mobility and time-dependent fluid flow are diminished, increasing the instantaneous contribution of the solid matrix (collagen + proteoglycan network) to load-bearing even where bulk compressive properties appear unchanged [48,49,236,238,239,240,241,242]. This load repartitioning elevates the local effective stiffness experienced by chondrocytes, promoting matrix microfissuring and maladaptive mechanotransduction in diffusion-limited microdomains. Aggrecan fragments and cell-derived DAMPs establish a locally self-reinforcing catabolic milieu driven by spatial metabolic heterogeneity rather than a uniform insult, which may preferentially affect the middle and deep zones of the knee and hip cartilage long before macroscopic changes are evident [195,196]. At the cellular level, reduced water mobility and altered ionic partitioning increase the demand for ATP-dependent ion transport (e.g., Na^+^/K^+^-ATPase) and perturb the pericellular ionic microenvironment, coupling mechanical alterations to metabolic stress and promoting localized bioenergetic deficits in diffusion-limited microdomains [235,239,243].

**Figure 7:**
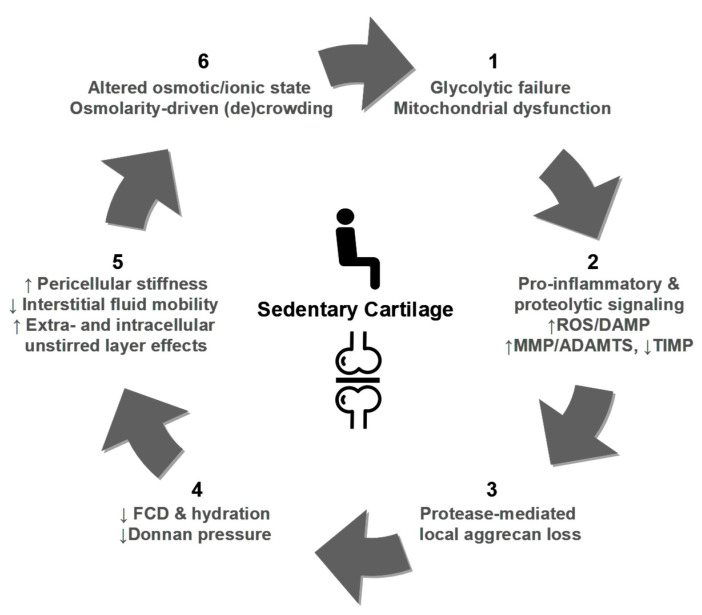
Schematic positive feedback loop linking diffusion-limited nutrient supply to focal cartilage catabolism. Diffusion limitation in thick and poorly mixed cartilage regions initiates a cascade of metabolic and biophysical events. (Node 1) Diffusion-limited nutrient supply causes glycolytic collapse and ATP depletion, leading to mitochondrial ROS production and DAMP release. (Node 2) These mitochondrial signals activate NF-κB/MAPK and inflammasome pathways and upregulate proteolytic enzymes (MMPs, ADAMTS), while impairing TIMP-mediated inhibition. (Node 3) Protease activity, together with undersulfation, drives aggrecan loss, lowering fixed-charge density and glycosaminoglycan content. (Node 4) Reduced fixed-charge density decreases tissue hydration and interstitial fluid mobility. (Node 5) Decreased fluid mobility increases the relative load borne by the solid pericellular matrix, promoting microfissuring and the generation of matrix-derived fragments that further amplify DAMP/protease signaling. (Node 6) Concomitant osmotic and ionic shifts cause cell swelling, reduced intracellular macromolecular crowding, and increased ATP demand for ion homeostasis, feeding back into glycolytic failure (return to node 1). Potential therapeutic interventions include improving solute transport (mechanical mobilization or intra-articular convection strategies), restoring pericellular osmolarity (osmolytes or intermittent loading), supporting mitophagy/antioxidant defenses, and selective protease inhibition (MMP/ADAMTS).

Recent experimental studies have shown that local extracellular osmolarity and the resulting changes in intracellular macromolecular crowding are potent regulators of chondrocyte metabolism and signalling [226,243]. In model systems, decreased molecular crowding (cell swelling associated with low extracellular osmolarity) increases chondrocyte sensitivity to pro-inflammatory stimuli and suppresses anabolic programs, whereas restoring physiological osmolarity renormalizes intracellular crowding, reverses glycolytic dysfunction, and reduces catabolic responsiveness. Such microenvironmental modulation plausibly links thickness-driven diffusion deficits (which perturb local ionic/osmotic balance and water content) to the intracellular energetic and transcriptional shifts that precede catabolism. Importantly, altered osmotic conditions and reduced intracellular crowding impair cell responsiveness to anabolic cues (e.g., TGF-β/BMP signaling) and shift transcriptional programs away from matrix synthesis, compounding the direct blockade of proteoglycan production [118,235,236,239,243,244,245,246,247,248]. Because osmolarity controls cell volume, dry-mass density, and macromolecular crowding—and thus proteostasis and signaling mobility—local osmotic shifts provide a concrete molecular mechanism by which modest diffusion deficits in thick cartilage can be amplified into focal energetic collapse, precipitating aggrecan loss, and establishing a self-reinforcing catabolic niche (Figure 7). These findings suggest that therapeutic strategies that restore or mimic healthy pericellular osmotic conditions (or otherwise preserve intracellular crowding) are candidate approaches to protect glycolytic flux, sustain anabolic signaling, and reduce early focal catabolic activation in diffusion-limited cartilage—hypotheses that now require systematic preclinical testing.

Together, these findings underscore that chondrocyte metabolism is heterogeneous across tissue microdomains rather than uniform. Local increases in effective pericellular stiffness and altered poroelastic response modify mechanotransduction pathways that control metabolism, gene expression, and matrix synthesis, biasing cells toward catabolic programs (e.g., upregulation of MMPs/ADAMTS and suppression of anabolic factors), thereby closing a feed-forward loop between metabolic heterogeneity, mechanical microenvironment, and matrix degradation [48,49,235,236,238,239,240,241,242]. Within the SCS framework, incipient, spatially heterogeneous stiffening of the ECM—which raises effective pericellular stiffness—interacts with diffusion-limited metabolism to generate microdomains at an elevated risk for early catabolic onset. Recognizing and mapping these transport–zonation–response interactions using spatial transcriptomics, depth-resolved mechanics, metabolic microsensors, and compositional imaging is essential to identify vulnerable niches and design targeted therapies that protect cartilage and delay OA onset.

### 9.3. Mechanoinflammatory Cascades and Circadian Dysregulation in Cartilage Catabolic Progression

Mechanical loading is a central regulator of cartilage homeostasis. Physiological cyclic loading promotes ECM synthesis, chondrocyte function, and convective transport of macromolecules that deliver anabolic factors and help clear metabolic waste products. However, excessive or dysregulated loading may be deleterious. Cyclic loading enhances convective transport but may also facilitate the penetration and accumulation of catabolic mediators in deeper zones when the transport–clearance balance is perturbed [249,250]. Cartilage structural anisotropy, from a collagen-rich superficial zone to a proteoglycan-dense deep zone, shapes stress distribution and zonal vulnerability [48,49]. The superficial layer, which endures the highest shear and compressive forces, is a common origin of matrix disruption and fragments (e.g., FN-fs, COMP, tenascin-C) [116,173,174]. Presumably, these MDPs act as DAMPs, engaging innate receptors (e.g., integrins such as α5β1 and Toll-like receptors TLR2/TLR4) to trigger cytokine release and initiate the mechanoinflammatory cascade characteristic of pre-OA [148,149]. Importantly, the amplitude and biological outcome of these responses depend on the exposure dose and timing (brief, high-intensity versus prolonged, cumulative loading) and are likely modified by the local metabolic state and circadian phase—factors that should be incorporated into experimental and translational designs.

Maroudas and colleagues [249] showed that cyclic compressive loading significantly increases convective transport of large solutes while having little effect on small nutrient supply. In human femoral-head cartilage plugs exposed to a simulated walking protocol (e.g., 2–8 MPa at 1 Hz), the convective transport of large solutes (e.g., serum albumin, 66.5 kDa) increased by approximately 30–100% under load, depending on the tracer size and experimental conditions [249]. These data indicate that mechanically driven interstitial fluid flow preferentially increases the penetration of biologically relevant macromolecules, such as COMP (~200 kDa), FN fragments (~50–200 kDa), and many cytokines (~8–60 kDa), from the superficial layer into the middle and deep zones, whereas small solutes (oxygen, glucose) remain diffusion-dominated. Consequently, cyclic mechanical loading creates a dynamic environment in which convection can deliver both anabolic and catabolic macromolecules into previously isolated zones, increasing the likelihood that ECM fragments and pro-inflammatory mediators will influence zonal cell responses in the articular cartilage.

**Figure 8:**
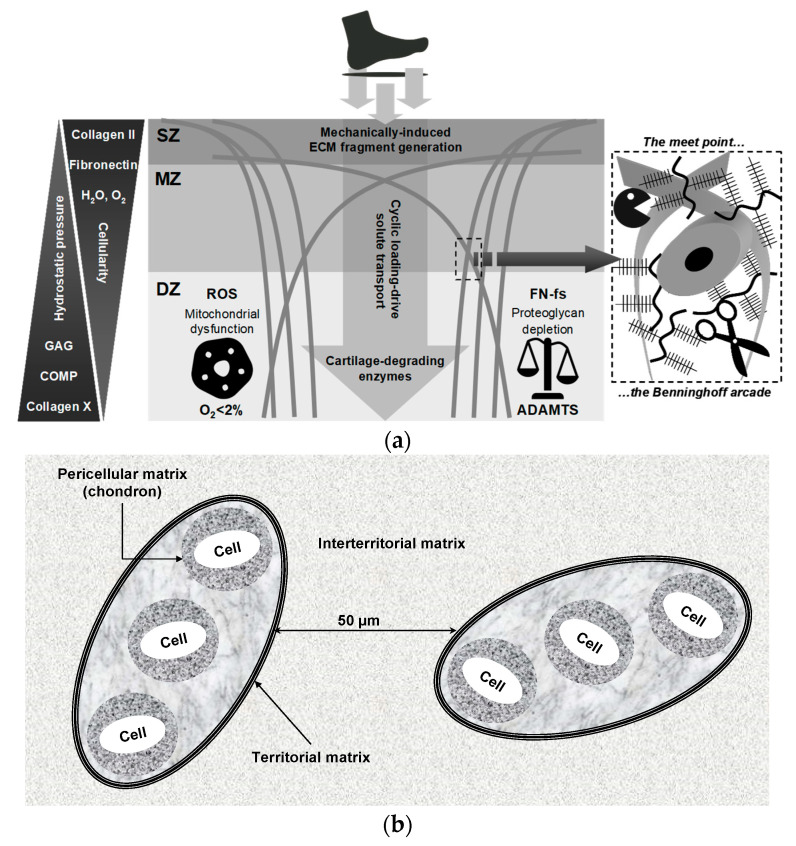
Cartilage under stress: Mechanically driven transport, inflammatory propagation, and circadian disruption in early OA. (**a**) Convective pumping and zonal exposure. Cyclic compression–decompression generates interstitial fluid flow that markedly enhances the convection of mid- and large-molecular-weight solutes (e.g., COMP, FN-fs, and cytokines), while small nutrients (oxygen, glucose) remain largely diffusion-limited. Cyclic loading increases the convective flux and effective permeability of the matrix, promoting deeper penetration of proteolytic fragments and pro-inflammatory mediators into the middle and deep zones. Accordingly, the deep zone of thick, load-bearing cartilage can be a critical vulnerability, where a concentrated “cocktail” of MDPs and cytokines drives local catabolic progression. These molecular and structural changes can convert transient osmotic or circadian perturbations into a persistent (“memory”) state that reinforces mechanical dysregulation and inflammation, thereby accelerating the transition from pre-OA to early OA. Understanding the intersection of transport dynamics, zonal architecture, and epigenetically reprogrammed cellular responses is therefore crucial for identifying intervention points that preserve metabolic flexibility, cartilage integrity and reduce the risk of OA development. (**b**) Paracrine contagion, osmotic collapse, and pre-OA thresholds. Early OA should be viewed as a spatial mosaic of subtle and localized metabolic imbalances rather than a uniform and tissue-wide degeneration. Individual chondrons act as semi-autonomous sensors (single-cell units ~10–20 µm) and chondron clusters (approximately 50–150 µm) that interpret biochemical and mechanical cues and generate spatially heterogeneous pro-inflammatory gradients. Short-range paracrine signaling between adjacent chondrons (and chondron clusters) and the convective influx of MDPs together sustain local cytokine exposure and induce MMP/ADAMTS expression, initiating focal ECM remodeling. Aggrecan depletion lowers fixed-charge density and Gibbs–Donnan swelling pressure, creating hypo-osmotic stress that impairs osmotic phase-resetting of chondrocyte clocks and represses genome-wide rhythmic gene expression. These spatially and temporally localized disturbances establish a self-reinforcing mechanoinflammatory loop that allows focal pre-OA foci to expand and coalesce into early stage OA. (Schematic not to scale). Abbreviations: COMP, cartilage oligomeric matrix protein; FN, fibronectin; MDP, matrix-derived degradation products; MMP, matrix metalloproteinase; ADAMTS, a disintegrin and metalloproteinase with thrombospondin motifs. Adapted from: [198,206,226,232,235,236,249]. See Table 1 and Table 2 for biomarkers and assays.

As shown in Figure 8a, interstitial fluid flow functions as a convective “pumping” mechanism that carries large solutes from the superficial layer into the middle and deep zones under the physiological cyclic loading. Repeated exposure of deeper-zone chondrocytes to pro-inflammatory mediators (for example, IL-1β and FN-fs) sustains the local induction of proteolytic enzymes, notably MMP-13 and ADAMTS-5, which progressively may erode matrix integrity even in regions of relatively higher density and lower permeability [166,167]. Because convection overcomes the diffusion limits for large macromolecules, this process concentrates proteolytic activity in structurally critical zones, driving matrix loss and impaired mechanical function. Specifically, I hypothesize that such redistribution of biochemical insults establishes a self-perpetuating mechano-inflammatory loop in which ECM fragmentation enhances mediator penetration, induces further protease expression, and stabilizes the chronic pre-OA state. In short, load-driven transport can propagate superficially initiated microdamage into deeper cartilage layers, converting focal surface injury into widespread, mechanically consequential degeneration. Empirical tests of this sequence require synchronized tracer/convection experiments, depth-resolved molecular assays, protease-blocking studies and linked mechanical endpoints.

Anisotropic diffusivity in joint cartilage, where larger solutes diffuse more readily along the collagen fiber orientation than across it, substantially shapes the spatial distribution of ECM fragments and pro-inflammatory cytokines. Superficial-zone chondrocytes respond to mechanotransduction and fragment–integrin signalling and produce cytokines such as IL-1β and TNF-α [166,167], and cyclic loading together with anisotropic transport drives their propagation into adjacent chondrons and vertically into the middle and deep zones [249]. As illustrated in Figure 8b and summarized in Table 4, this lateral and vertical spread stimulates localized upregulation of matrix-degrading proteases (e.g., MMP-13 and ADAMTS-5) and early ECM remodeling [206]. Concurrent aggrecan loss reduces fixed-charge density and Gibbs–Donnan swelling pressure, producing hypo-osmolar stress that impairs circadian entrainment and genome-wide rhythmic expression in chondrocytes [226,236]. Collectively, anisotropic transport, paracrine propagation (which may involve extracellular vesicles and microRNAs), and osmotic-driven clock disruption form a spatially and temporally self-reinforcing loop of ECM fragmentation, cytokine signalling, and proteolysis that potentially accelerates the progression from pre-OA to early OA. However, direct evidence for vesicle/miRNA mediation in vivo remains limited, warranting targeted validation.

Putatively, the transition to early OA is marked by a critical, empirically determinable threshold at which adaptive, reversible changes give way to irreversible structural failure [70]. Beyond this point, homeostatic repair mechanisms are overwhelmed, structural integrity deteriorates, and progressive cartilage loss occurs. A key determinant of this threshold is damage to the collagen network, notably the disruption of Benninghoff’s arcade-like collagen architecture in the middle and deep zones, which irreversibly compromises load distribution and biomechanical competence [73,118,120]. In the early stages, aggrecan depletion lowers osmotic swelling and cartilage resilience, exposing collagen fibers to increased mechanical and enzymatic insults and accelerating collagen fragmentation. Operationalizing this tipping point requires validated collagen-sensitive measures (for example, UTE/T2* MRI, collagen-hybridizing peptide assays, and circulating collagen cleavage products such as CTX-II or Coll2-1), depth-resolved mechanical testing, and longitudinal outcome data. Recognizing and measuring this structural threshold defines a practical window for preventive strategies aimed at preserving the collagen scaffold and for trials that test whether intervention before threshold crossing prevents disease progression. Importantly, the threshold is probabilistic and modified by age, biomechanics, and comorbidity; thus, its clinical definition requires prospective longitudinal validation.

Empirical data support this conceptualization. Previous studies have indicated that proteoglycan depletion correlates with progressive collagen network damage and that internal matrix disruption can accelerate further loss of proteoglycan and collagen integrity [127]. Unlike other matrix components, collagen exhibits an extremely slow turnover, making collagen damage long-lived and difficult to reverse [73,120]. Progressive collagen degradation compromises the ability of the tissue to effectively distribute mechanical loads, increasing cellular stress, apoptosis, matrix breakdown, and chronic inflammation, which drive cartilage loss and subchondral remodeling, producing the self-perpetuating joint degeneration characteristic of early OA. Framed within this expanded mechanoinflammatory model, pre-OA is spatially localized and temporally dysregulated: loss of osmotic swelling pressure and associated circadian disruption further diminish adaptive repair capacity [226,236]. Crucially, these observations define a pre-radiographic therapeutic window in which targeted early interventions—load modification and neuromuscular prehabilitation, metabolic support, osmotic/restorative approaches, chronopharmacologic scheduling, selective anti-protease interventions, and agents that stabilize or protect the ECM (some investigational)—may restore rhythm and matrix homeostasis and prevent progression to established OA. The predictive value, timing, and generalizability of such interventions require prospective validation in biomarker-enriched longitudinal cohorts and randomized trials.

## 10. A Tetrahedral Framework for Pre-OA: Integrating Mechanical, Structural, Behavioral, and Circadian Drivers of Early Joint Degeneration

**Figure 9:**
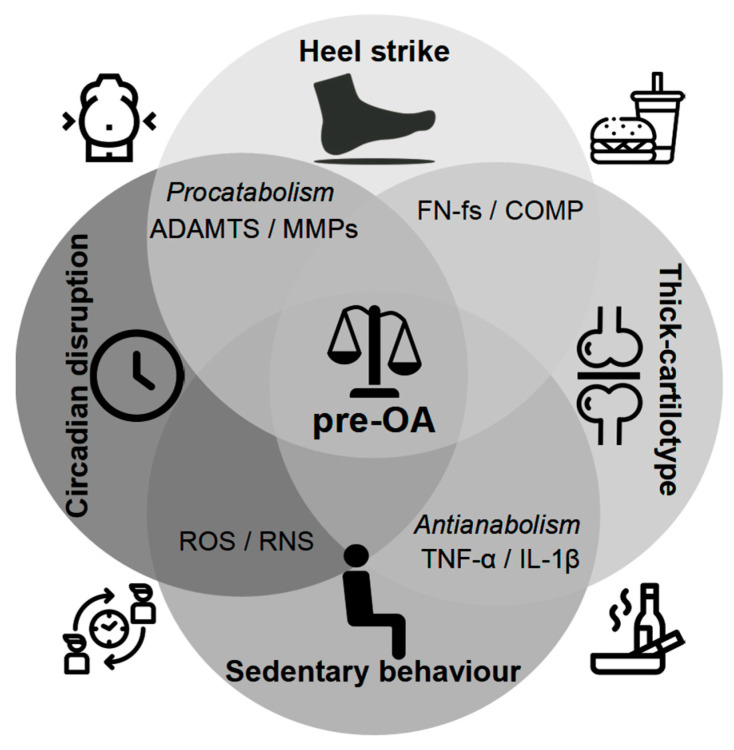
Tetrahedral framework for early cartilage degradation in the 21st century, providing a rationale for using subclinical biomarkers to inform stratified intervention strategies. A multifactorial, cartilage-centric model in which four interdependent domains converge to destabilize matrix homeostasis, driven in part by a sustained chondrocyte metabolic imbalance, and thereby generate a mechanistic pre-OA endotype: (1) mechanical stress—repetitive high-rate transients (HST; peak vGRF, loading rate); (2) cartilage thickness—the thickness of healthy, mature native tissue (e.g., knee and hip cartilage)—increases diffusion distances and thereby constrains nutrient influx and waste clearance (the “limiting-depth” effect) in deeper zones, creating metabolically vulnerable regions; (3) sedentary behavior—reduced cyclical flexion–extension joint motion and attenuated mixed (convective–diffusive) synovial transport, which together compromise lubrication and decreased matrix turnover; and (4) circadian rhythms—disruption of cartilage clock-regulated anabolic and catabolic programs. The schematic (converging circles meeting at a central “pre-OA” node) emphasizes cumulative and synergistic effects: repetitive mechanical transients initiate microtrauma and convective transport of MDPs, cytokines, and reactive species (including oxygen and nitrogen derivatives) into deeper zones; relatively thick cartilage impairs nutrient diffusion/delivery and waste clearance; prolonged sedentary behavior impairs cartilage lubrication and disrupts ECM remodeling by reducing cyclical mechanical stimuli; and circadian misalignment deranges the temporal regulation of anabolic repair programs in cartilage. These spatiotemporally overlapping processes interact with neuromuscular function/control, early metabolic perturbations, and local inflammatory responses, whereas systemic metabolic factors (for example, obesity/metabolic syndrome) act as amplifiers, driving a tipping point toward catabolism and accelerated matrix loss. Mapping the timing of intrinsic cartilage clocks and mechano-metabolic programs is therefore essential to identify temporal windows for targeted multimodal early intervention. Adapted from: [209,213,217,231,251,252,253,254,255,256,258]. Abbreviations: ADAMTS, a disintegrin and metalloproteinase with thrombospondin motifs; COMP, cartilage oligomeric matrix protein; FN-fs, fibronectin fragments; IL-1β, interleukin-1 beta; MDP, matrix-derived degradation products; MMPs, matrix metalloproteinases; pre-OA, pre-osteoarthritis; RNS, reactive nitrogen species; ROS, reactive oxygen species; TNF-α, tumor necrosis factor-alpha.

OA should not be viewed as a single end state but as a temporally and spatially heterogeneous continuum arising from the interaction of biomechanical, biochemical, genetic–circadian, and environmental perturbations. Because established OA is largely irreversible, these interacting processes frustrate the straightforward translation of bench research to clinical applications. Emerging evidence, however, supports a clinically silent and molecularly distinct pre-OA phase that offers the most promising window for preventive action. To operationalize this latent stage, I propose a cartilage-centric pre-OA endotype—a mechanistically defined allostatic state of the joint matrix—specified by four interdependent domains (Figure 9): dynamic mechanical loading (e.g., HST characteristics such as peak vGRF and loading rate), cartilage microarchitecture—regional, quantitative MRI biomarkers and acquisition protocols sensitive to ultra-early deep-zone ECM alterations (e.g., quantitative T2/T2*, T1ρ, dGEMRIC, UTE or sodium imaging, and high-resolution thickness mapping), with pre-specified ROI definitions and minimal detectable-change criteria, sedentary behavior (accelerometry and standardized questionnaires), and circadian regulation (tissue and physiologic clock markers). Framing pre-OA as a defined endotype rather than a nonspecific precursor provides a mechanistic scaffold for early detection, targeted phenotyping, and precision-tailored prevention aimed at interrupting maladaptive matrix remodeling before irreversible decline. This tetrahedral model highlights how structural (tissue thickness), mechanical (loading transients), behavioral (sedentary time), and temporal (circadian timing) inputs converge to destabilize cartilage homeostasis, while accommodating joint-specific variability and secondary amplifiers—malalignment, obesity, metabolic dysfunction, smoking, poor diet, and psychosocial stress—that shift individuals’ trajectories along the OA continuum [21,251,252,253,254,255,256,257]. Mapping this mechanistic scaffold onto quantifiable metrics and modifiable interventions could create a practical framework for early detection, individualized risk stratification, and preventive therapy.

In otherwise “healthy” but inactive individuals, SCS can be understood as a systems-level manifestation of the sedentary–thickness axis that preferentially stresses deep-zone cartilage and establishes a locally adverse metabolic state [195,196]. Mitochondrial dysfunction and impaired autophagy promote early aggrecan loss, whereas recurrent mechanical transients drive the convective transport of MDPs, cytokines, and other pro-inflammatory mediators from the superficial layer into the tissue interior, amplifying proteoglycan degradation and perturbing osmotic homeostasis. These overlapping spatiotemporal insults may increase catabolic signaling and disrupt the circadian program of joint cartilage, diminishing mechanically induced anabolic responses and impairing the timing and amplitude of clock-regulated anabolic genes in sedentary individuals [226,235]. In this context, where the pro-degenerative microenvironment renders deep cartilage layers particularly susceptible, I hypothesize that genetic variation at core-clock loci or in cartilage-specific regulatory elements may modulate clock amplitude or phase (for example, via BMAL1/CLOCK, PER/CRY, and NR1D1 pathways), biasing the tissue toward relative upregulation of catabolic effectors (MMP13, ADAMTS5), and suppression of anabolic circuits (SOX9, COL2A1, ACAN, TGF-β/SMAD). The downstream effects are impaired proteoglycan and collagen synthesis, shifted autophagy–repair balance, and insufficient microdamage restoration, producing a molecularly abnormal but clinically silent pre-OA endotype, which is most sensitively revealed by time-series transcriptomics, longitudinal biomarker trajectories, and compositional MRI [69,84,85]. This integrative framework is supported by cartilage-specific Bmal1 loss-of-function experiments and observations of reduced BMAL1 expression and disrupted circadian transcriptional rhythms in the human cartilage. Functional genomic analyses have further indicated that many OA risk variants influence disease susceptibility by altering the regulatory elements that modulate tissue-specific transcriptional responses [20,22,213,218]. Together, this model supports translational approaches—chronotherapy, clock-modulating small molecules, and time-targeted prehabilitation—to restore anabolic rhythmicity and reduce the risk of progression from molecular pre-OA to established (early) OA. Importantly, these genetic and environmental interactions are probabilistic risk modulators that require functional genomic and longitudinal validation.

If the tetrahedral model reliably captures a clinically meaningful biology, it has three immediate implications. First, early detection must move beyond radiography and symptoms toward multimodal pheno-endotyping that maps when (temporal emergence) and where (spatial locus, intra- and inter-joint) metabolic dysregulation occurs. Such pheno-endotype should integrate time-series molecular profiling, serial serum/urine biomarker panels, mechanophenotyping (force plates, instrumented treadmills, wearable sensors), and high-resolution compositional MRI in research cohorts to establish parsimonious core batteries for broader clinical implementation [4,14,15,26,67,68,259]. Second, the intervention design should be mechanism-matched and multifaceted: simultaneously correcting mechanical drivers (alignment, orthoses, gait retraining), mitigating systemic amplifiers (weight loss, metabolic optimization), reducing sedentary burden (activity pattern modification, timed loading), and re-entraining circadian anabolic programs (chrono-exercise, sleep hygiene, pharmacologic clock modulation) [209,213,251,260,261]. Third, the model defines the testable translational pathways. Biomarker-enriched trials and adaptive designs should evaluate chronotherapy, clock-modulating small molecules, targeted prehabilitation, and chondroprotective agents using quantitative imaging and mechanophenotyping as primary or intermediate end-points [262,263]. To make these strategies equitable and scalable, the parallel development of low-cost surrogate biomarker panels for primary care is essential, along with early regulatory engagement to qualify intermediate biomarkers for use in trials.

Realizing this agenda will require coordinated multidisciplinary collaboration to translate distinct pathobiological mechanisms into ultra-early precision and preventive medicine. Transitioning OA care from late-stage symptom management to proactive prevention depends on the prospective validation of early-stage molecular and imaging biomarkers, development of standardized mechanophenotyping protocols, and scalable interventions that target tetrahedral drivers and their systemic amplifiers [264]. Central to implementation is incorporating the cartilage-centric pre-OA endotype in clinical workflows to enable robust risk stratification, targeted patient selection, and adaptive chondroprotective algorithms informed by multimodal pheno-endotyping (time-series transcriptomics, serial fluid biomarkers, quantitative MRI) and mechanistic functional assays [259]. Despite the current challenges in the field, operationalizing the tetrahedral framework through biomarker-enriched early phase and adaptive trials can enable the rigorous evaluation of chronobiologic, mechanical, metabolic, and chondroprotective strategies using quantitative imaging and mechanobiologic endpoints, with the explicit aim of detecting, stabilizing, and reversing matrix dysfunction before irreversible structural failure [26].

## 11. Targeting Pre-OA: Multimodal Strategies for Chondro-, Mechano-, and Osmoprotection

### 11.1. The Cornerstones of Pre-OA Intervention: Modulating Cartilage Metabolic Dysregulation

OA is a complex health challenge that requires multidisciplinary strategies, but prevention at the pre-OA stage offers a tractable and scalable opportunity. Pre-OA is defined here as the state in which the articular cartilage remains macroscopically intact yet exhibits measurable perturbations in ECM homeostasis, catabolic signalling, and cellular metabolism—an ultra-early metabolic imbalance of cartilage cells that may be amenable to preventive measures. Similarly to daily oral hygiene preventing tooth decay, consistent, targeted prevention at the pre-OA stage could reduce the need for later invasive treatments [21,113]. Successful prevention therefore aims to restore chondrocyte homeostasis by normalizing bioenergetics, preserving autophagic flux, limiting protease activity, and shifting ECM turnover toward repair. Practically, this suggests a multipronged toolkit: metabolic modulators that support cellular energetics (for example, AMPK/PGC-1α pathway activation or NAD^+^-boosting strategies—investigational), selective anti-catabolic agents and localized protease inhibitors, anabolic stimuli (exercise, timed loading, growth-factor approaches), and antioxidant/mitochondrial-protective therapies [265]. Given the multiplicity and functional redundancy of early catabolic, inflammatory, and metabolic circuits in pre-OA, single-agent interventions are unlikely to produce sustained modifications in the disease. Instead, rationally constructed combination regimens that co-target early matrix fragments, incipient inflammatory and oxidative pathways, matrix-degrading enzymes, chondrocyte bioenergetic dysfunction, and maladaptive biomechanical drivers delivered and timed using biomarker-guided stratification provide a mechanistically coherent and pragmatically testable strategy to arrest or substantially slow progression in the ultra-early stage [265]. Equally important are non-pharmacologic measures, such as neuromuscular conditioning, weight and nutritional management, smoking/alcohol avoidance, and sleep optimization, which modify the mechanical and systemic metabolic milieu. Because pre-OA metabolic dysregulation arises from temporally overlapping, spatially heterogeneous, and mechanistically redundant processes, biomarker-guided stratification followed by rationally designed combination therapies—initiated during the ultra-early stage—is essential to arrest or substantially slow the pre-disease progression. Below, I synthesize the biological rationale, mechanistic evidence, candidate interventions, and implementation challenges, and propose a staged translational roadmap for testing prophylactic strategies in well-defined pre-OA cohorts. If validated prospectively in biomarker-enriched cohorts, multimodal, time-aligned chondroprotective strategies could reframe OA from an inevitable late-life condition into a largely preventable disorder, substantially reducing the need for invasive interventions and preserving mobility and quality of life at the population scale.

### 11.2. Integrating the CADENCE Chrono-Framework into OA Prevention: Rationale and Translational Roadmap

**Figure 10:**
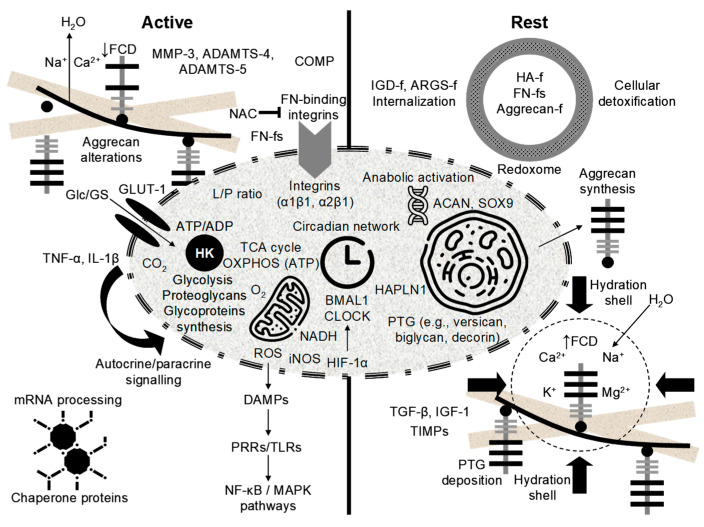
Chondron functional states: active (catabolic) versus resting (maintenance). Left (active): enhanced ECM breakdown (e.g., aggrecanolysis, ARGS neo-epitope), increased transmembrane nutrient/ion flux to support heightened biosynthetic/energetic demand, amplified mechanotransduction and pro-inflammatory signalling (IL-1á/TNF-á → MAPK/NF-κB) and mitochondrial stress (↑ mtROS, dysregulated ATP). Right (resting): predominant biosynthetic output and regulated matrix turnover (SOX9, ACAN, COL2A1), preserved ionic/osmotic homeostasis (fixed-charge density), intact autophagic and endo-lysosomal processing of matrix fragments, and efficient mitochondrial function. Solid arrows denote material flux (nutrients, matrix-derived fragments) and regulatory signalling. The panels emphasize the dynamic, reversible balance between these programs and their modulation by physiological mechanical loading and circadian timing. Abbreviations: ACAN, aggrecan; ADAMTS-4, a disintegrin and metalloproteinase with thrombospondin motifs 4; ADAMTS-5, a disintegrin and metalloproteinase with thrombospondin motifs 5; ADP, adenosine diphosphate; ARGS-f, aggrecan ARGS neo-epitope fragment; ATP, adenosine triphosphate; BMAL1, brain and muscle ARNT-like 1; CLOCK, circadian locomotor output cycles kaput; COL2A1, type II collagen alpha 1 chain; COMP, cartilage oligomeric matrix protein; DAMPs, damage-associated molecular patterns; FCD, fixed-charge density; FN-fs, fibronectin fragments; Glc, glucose; GS, glucosamine sulfate; GLUT-1, glucose transporter 1; HAPLN1, hyaluronan and proteoglycan link protein 1; HA-f, hyaluronic acid fragments; HIF-1α, hypoxia-inducible factor 1 alpha; IGD-f, interglobular domain fragments; IGF-1, insulin-like growth factor 1; iNOS, inducible nitric oxide synthase; L/P ratio, lactate/pyruvate ratio; MAPK, mitogen-activated protein kinase; MMP-3, matrix metalloproteinase-3; mtROS, mitochondrial reactive oxygen species; NADH, nicotinamide adenine dinucleotide; NAC, N-acetyl cysteine; NF-κB, nuclear factor kappa B; OXPHOS, oxidative phosphorylation; PRRs, pattern recognition receptors; PTG, proteoglycan; SOX9, SRY-box transcription factor 9; TCA cycle, tricarboxylic acid (Krebs) cycle; TGF-β, transforming growth factor beta; TIMPs, tissue inhibitors of metalloproteinases; TLRs, toll-like receptors; TNF-α, tumor necrosis factor alpha.

Circadian clocks are increasingly recognized as critical regulators of cartilage homeostasis, and chondrocyte function is temporally structured across a 24-h day [217]. Proteomic profiling in animal models has revealed robust daily oscillations in the pathways governing ATP production, glucose metabolism, cytoskeletal remodelling, and ECM synthesis, implying discrete windows of vulnerability and repair (Figure 10; Table 5). Although these data provide strong biological rationale for time-targeted interventions, translation is hindered by the lack of phase-resolved proteomic atlases in human cartilage. Establishing a human circadian atlas by integrating synchronized ex vivo cartilage explants, iPSC-derived chondrocyte organoids, spatial/time-series transcriptomics, and serial serum/synovial proteomics paired with wrist actigraphy (continuous rest–activity/sleep–wake monitoring) would constitute a timely near-term objective that could be prioritized within broader chrono-research initiatives, such as the Chondroprotection Advanced through Deliberate Exercise and Networked Circadian Engagement (CADENCE) framework [265]. These datasets will identify human-relevant phases and markers that can be used to design and pilot chrono-interventions (timed exercise, chrono-dosing, and clock-modulating agents) in biomarker-enriched cohorts.

The CADENCE chrono-framework proposes a principle-based chrono-strategy that integrates circadian biology with preventive measures for early-stage OA, partitioning the 24-h day into four functional windows—protection, pro-anabolism, hygiene, and restoration—and suggests the timing of nutritional, pharmacological, and mechanical inputs to align with chondrocyte activity and matrix turnover phase [265]. For example, the “protection” (high-activity) window is intended to promote optimal cartilage lubrication and nutrient supply and to prioritize temporally targeted chondroprotective interventions and controlled joint motion aimed at mitigating early inflammatory activation and matrix-degradative cascades; the “pro-anabolism” window leverages chronoexercise and metabolic cofactors, together with mechanotransductive signals that stimulate matrix synthesis; the “hygiene” window emphasises metabolic waste clearance to preserve cartilage homeostasis; and the “restoration” window focuses on nocturnal cellular repair, tissue rehydration, and circadian-entrained regenerative processes to support long-term cartilage health. CADENCE is hypothesis-generating and is not a prescriptive clinical regimen until human phase-resolved validation is completed; rather, it provides a testable roadmap to define optimal timing, quantify inter-individual variability (chronotype), and evaluate whether temporally targeted measures improve biological and clinical outcomes in OA prevention [265].

Phase-resolved proteomics offers mechanistic insights into CADENCE but should be treated as provisional until human validation is obtained. In model systems, the early active interval is enriched for proteins and chaperones linked with ATP synthesis and energetic support (e.g., HSPA9, HSP90 family, MATN1, and PLOD2), consistent with increased cellular capacity for biosynthesis and matrix turnover (see Table 5, for details) [217]. Therefore, this window is well suited to strategies that synchronize nutrient provision with chronoexercise to enhance the delivery of anabolic precursors [265]. Simultaneously, mechanical loading releases ECM fragments that stimulate cytokine signalling and accelerate catabolic turnover, and ROS generated downstream of FN-fs and other DAMPs can amplify fragmentation-driven chondrolysis [91,148,149,162,164,165,166,167,168,169,178,185,186]. Consequently, the early active window is a logical time to test combined approaches pairing physical activity/exercise with chondroprotective or antioxidant co-therapies that suppress catabolic signalling and supply anabolic substrates [265,266,267]. Candidate agents (to be considered investigational for this application) include glucosamine sulfate and N-acetylcysteine (NAC), which merit rigorously controlled, time-specific trials rather than off-label clinical endorsement [113,268,269,270,271,272,273,274]. The late active phase, characterized by increased glucose uptake and proteostasis proteins (SLC2A1/GLUT1, PKM, and molecular chaperones) [217], may favor resistance and functional exercise, together with interventions that sustain proteostasis and reduce proteotoxic stress. Complementing redox control, dynamic compression modulates local Na^+^ and water distribution across stiffness gradients, altering pericellular ionic interactions that regulate growth factor binding and activation (notably TGF-β in aggrecan-rich, highly fixed-charge regions), and produces a convective “pumping” effect that enhances the delivery of anabolic precursors into cartilage [265]. Mechanistically motivated strategies should be evaluated using assays that capture convective delivery (e.g., tracer uptake, sodium MRI), proteolytic activity, and rhythmic biomarker changes, using short, biomarker-driven pilot trials to determine which time-dependent combinations warrant larger adaptive testing.

The rest phase is associated with proteasomal and collagen-stabilizing programs (PSMB/PSMD family, PLOD1/2, SERPINE1), consistent with the daily structural rearrangement and damaged protein clearance observed in model proteomes [217]. This phase coincides with intensified interactions between ECM fragments and chondrocyte receptors: small aggrecan fragments (often HA-bound) may be internalized via CD44-dependent endocytosis and routed to lysosomes, whereas larger, highly charged glycosaminoglycan aggregates remain extracellular [275,276,277]. In experimental systems, local accumulation of ECM fragments and metabolic byproducts (lactate, CO_2_) can produce mild acidification that enhances ligand availability for integrins, TLRs, and inflammasomes and modulates extracellular protease activity, biasing signaling toward sustained NF-κB/MAPK/MyD88 cascades and upregulation of MMPs, ADAMTS, and inflammatory cytokines [12,92,149,178,278,279]. Such pH- and fragment-sensitive mechanisms support the concept of chondrohygienic strategies—non-invasive measures that restore fluid exchange and facilitate fragment clearance (timed mechanical loading, synovial-promoting activity, and hydration)—as testable interventions to limit sustained catabolism [265]. The late rest phase, associated with peak translational activity, further emphasizes the potential role of sleep-dependent restoration and interventions that support ribosomal function, proteoglycan synthesis and incorporation into the ECM, and tissue rehydration in cartilage recovery (Figure 10; Table 5). Because these assignments are largely derived from rodent proteomes, they remain provisional, and rigorous phase-resolved human cartilage studies and controlled clinical trials are required. Accordingly, CADENCE should be viewed as a hypothesis-generating scaffold rather than a prescriptive regimen—one that reframes circadian biology into a translational roadmap for OA prevention while emphasizing the urgent need for rigorous human validation [265,267].

### 11.3. Should Prophylactic Pharmacotherapy Be Considered in the Pre-OA Window? Opportunities, Challenges, and Translational Potential

The lack of approved disease-modifying osteoarthritis drugs (DMOADs) reinforces the rationale for prevention over late-stage rescue paradigms. Accordingly, the pre-OA window—when the cartilage remains macroscopically intact but shows measurable disruptions in ECM homeostasis and cell signalling—represents a biologically plausible opportunity for prophylactic pharmacotherapy. At this stage, the overall burden of catabolic mediators is relatively modest (e.g., low-level increases in ECM fragments, TNF-α/IL-1β, and MMPs/ADAMTS), suggesting that careful pharmacologic modulation could interrupt a self-amplifying degradation (aggrecanolysis) cascade before irreversible fibrillation occurs (Figure 6; Table 2). Natural bioactive compounds, such as curcumin, resveratrol, ascorbic acid, and glucosamine, are biologically active, naturally derived agents under active investigation for potential roles in clinical and preventive medicine and therefore represent plausible low-risk candidates for pre-OA modulation [113,265,268,269,270,271,272,273,280,281,282,283,284,285,286]. For example, Mattiuzzo et al. [283] demonstrated that low concentrations of D-glucosamine, particularly when combined with antioxidant compounds, can attenuate inflammatory signalling and increase collagen II and aggrecan expression in human chondrocyte cultures, providing mechanistic support for evaluating low-risk prophylactic strategies while underscoring that such promising preclinical findings require clinical validation of both efficacy and long-term safety in asymptomatic populations. This rationale supports a two-stage approach: (1) pragmatic evaluation of low-risk, broadly applicable chondroprotective agents with well-characterized safety profiles (e.g., rigorously standardized glucosamine sulfate formulations, selected antioxidant, or metabolic modulators) in biomarker-enriched prevention trials; and (2) judicious use of targeted mechanism-based inhibitors (e.g., ADAMTS/MMP antagonists) reserved for individuals at high probability of progression, ideally delivered locally to minimize systemic exposure [165,272,273,287,288,289]. Importantly, efficacy data for often considered benign nutraceuticals or “natural” chondroprotective candidates are mixed, and targeted protease inhibitors have historically raised safety or specificity challenges [267,273,284,286]. Therefore, all pharmacologic prophylaxis should be evaluated in randomized biomarker-guided trials with pre-specified intermediate imaging and molecular endpoints, robust safety monitoring, and explicit ethical safeguards for treating largely asymptomatic participants. Early regulatory consultation to qualify intermediate endpoints and cost-effectiveness modeling to assess population-level benefits will be critical to any successful prevention program.

**Figure 11:**
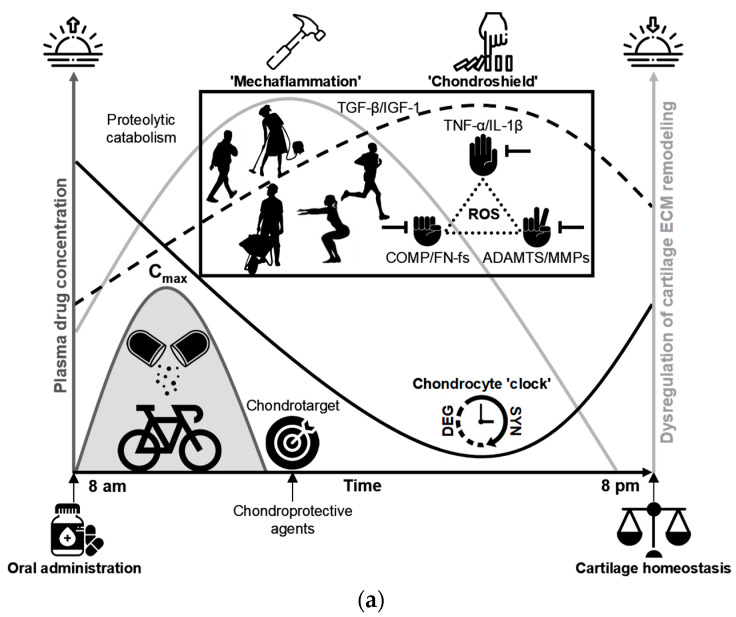

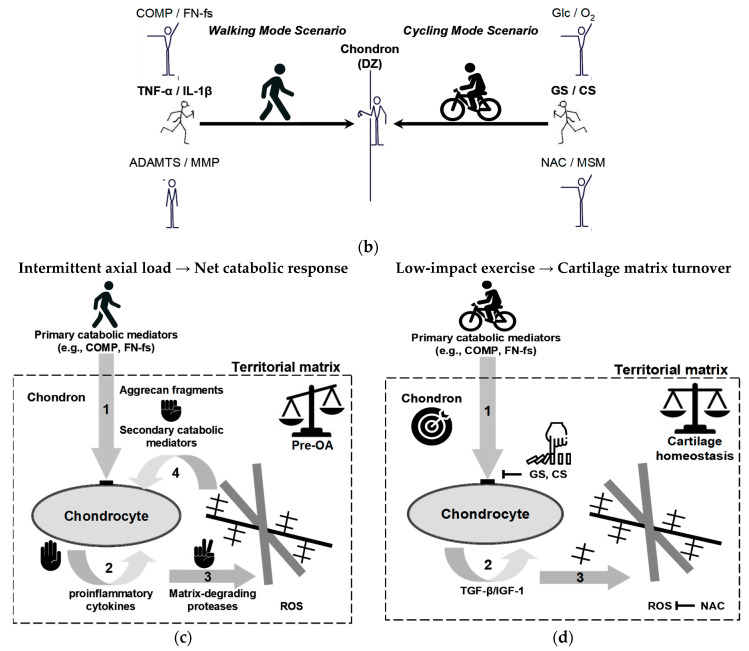
Integrative preventive model targeting early metabolic imbalance of hyaline cartilage in pre-OA. The panels illustrate how combining lifestyle adjustments with targeted therapeutics can potentially modulate intra-cartilage transport and slow the progression from ultra-early biochemical imbalance to structural degeneration. (**a**) Schematic representation of a multimodal preventive intervention combining timed low-impact exercise (chrono-exercise; bicycle icon), pharmacological chondroprotection, and lifestyle measures to optimize intra-cartilage transport and limit catabolic cascades. Because synoviocytes secrete approximately 10× more HA than chondrocytes and cyclic motion has been reported to nearly double synovial HA secretion, timed low-impact exercise is proposed to augment synovial HA delivery to the joint surface, improve superficial-zone tribology, and thereby reduce mechanically induced ECM fragment release and downstream pro-inflammatory responses, synergizing with pharmacological and lifestyle interventions to slow the progression from ultra-early biochemical imbalance to structural degeneration. (**b**) “Handkerchief-game” analogy: opposing “teams” of pro-catabolic factors (matrix fragments, cytokines, degradative enzymes) and chondroprotective agents compete to reach chondrons across cartilage depth, from superficial to deep zones; biomechanical regimes can influence which team predominates by shaping transport dynamics. (**c**) Weight-bearing walking produces convective “pumping” that accelerates the penetration of large pro-catabolic species (e.g., FN-fs, COMP, TNF-α, IL-1β, MMP-3, ADAMTS-4/5) into deeper zones, concentrating degradative signals at the chondron and promoting matrix breakdown. Early time-targeted measures that limit aggrecanolysis or suppress nascent inflammatory signalling could plausibly disrupt this self-amplifying cascade and reduce the risk of irreversible fibrillation and the incipient loss of load-bearing capacity. (**d**) Cycling (low-impact, high-frequency joint motion) reduces repetitive impact while effectively agitating synovial fluid, potentially enhancing the diffusion of small protective molecules into cartilage (examples include glucosamine sulfate—investigational for prevention) while relatively limiting the convective entry of large degradative species, thereby favoring pericellular preservation. These considerations further imply that concurrently addressing multiple treatment targets—for example, combining biomechanical modulation, inhibition of early matrix-degrading enzymes, suppression of nascent inflammatory signalling, reduction in oxidative stress, and support of anabolic growth-factor pathways—may be essential to halt or substantially slow pre-disease progression in pre-OA by interrupting parallel, self-amplifying pathogenic loops rather than relying on single-target interventions alone. Together, the panels illustrate how specific biomechanical regimes can modulate intra-cartilage transport of key molecular classes and support a combined pharmacological/non-pharmacological lifestyle-medicine strategy to preserve cartilage homeostasis in pre-OA. Abbreviations: ADAMTS, a disintegrin and metalloproteinase with thrombospondin motifs; COMP, cartilage oligomeric matrix protein; CS, chondroitin sulfate; DZ, deep zone; ECM, extracellular matrix; FN-fs, fibronectin fragments; Glc, glucose; GS, glucosamine sulfate; IGF-1, insulin-like growth factor 1; IL-1β, interleukin-1 beta; IL-6, interleukin-6; MMPs, matrix metalloproteinases; MSM, methylsulfonylmethane; NAC, N-acetylcysteine; pre-OA, pre-osteoarthritis; ROS, reactive oxygen species; TGF-β, transforming growth factor-beta; TNF-α, tumor necrosis factor-alpha.

Mechanistic and chronobiological data, largely from ex vivo and animal studies, support two interacting principles that should guide timed prophylaxis: (1) repetitive mechanical loading profoundly influences intra-tissue transport (convective “pumping” and diffusion) [249,250], and (2) circadian organization imposes predictable temporal peaks and valleys in chondrocyte activity [217,226,229]. Early cartilage catabolism is driven by loading- and inflammation-linked upregulation of degradative enzymes and cytokines (Figure 11a). Selective inhibition of key nodes, such as aggrecanolysis, may reduce irreversible matrix loss [88]. Chronobiology refines this picture by identifying reproducible time windows linked to daily activities (e.g., ambulation) when pro-catabolic signals and MDPs peak, thereby creating predictable vulnerability periods during which aligned pharmacotherapy could have an outsized impact [266]. Translational strategies should therefore integrate pharmacology, biomechanics, and chronobiology. Chronopharmacology synchronizes therapeutic delivery to coincide with activity- or circadian-driven catabolic peaks, maximizing on-target exposure while minimizing total dose, whereas chronoexercise uses controlled, low-impact, individualized activity (e.g., cycling) to promote the diffusive transfer of small chondroprotective molecules from synovial fluid into deeper cartilage layers, limit convective transport, and avoid provoking physiological damage [265,266,267]. As illustrated by the handkerchief-game analogy (Figure 11b–d), these competitive transport dynamics make the timing and mode of activity active potential determinants of whether protective or destructive molecules reach deep chondrons; combined, coordinated, timing-sensitive multimodal interventions that (i) attenuate catabolic peaks, (ii) deliver protective agents at key times and depths, or (iii) both, are the most likely to shift the balance toward cartilage preservation. Any chrono-intervention program must prioritize safety and individualization and be driven by human validation of timing and transport in pilot tracer and biomarker studies.

Translational implementation requires precise risk stratification, rigorous early mechanistic studies, and explicit attention to ethical, safety, and regulatory expectations. Initial clinical development should focus on discrete, high-risk cohorts (e.g., well-characterized post-traumatic injury cohorts and sedentary individuals with compositional MRI/biomarker evidence of pre-OA), where the at-risk window is temporally bounded and progression rates facilitate mechanistic and clinical inference [290]. Drug development should begin with short, randomized, biomarker-driven proof-of-mechanism studies that integrate synovial and systemic pharmacokinetics, demonstrate target engagement (validated molecular biomarkers), and identify compositional MRI endpoints. Chondroprotective agents demonstrating favorable exposure and biomarker effects should advance via adaptive, biomarker-enriched trial designs (for example, response-adaptive randomization and enrichment) to larger prevention trials that use co-primary or hierarchical endpoints linking early mechanistic changes to clinically meaningful outcomes (for example, time to persistent symptoms or validated structural surrogates) [26]. Because long-term prophylaxis in largely asymptomatic individuals raises safety, cost, and equity concerns, trigger-based strategies (biomarker- or event-initiated therapy), prioritization of agents with favorable safety profiles for broad use, and early engagement with regulators and payers to define acceptable surrogate endpoints and reimbursement pathways are necessary. Key knowledge gaps, including quantitative human synovial and intra-cartilage pharmacokinetics, comparative transport under different loading modalities, circadian characterization of human cartilage catabolic peaks, and durability/safety data, should be prioritized to de-risk translation. If mechanistic studies consistently confirm favorable intra-tissue exposure, target engagement, and tolerability, integrated chronotherapeutic strategies merit rigorous testing to determine whether early prophylaxis can meaningfully and durably alter the pre-OA trajectory [217].

Priority should be given to prevention-focused research in the preclinical stage of knee and hip OA, where emerging evidence suggests the highest likelihood of durable benefits. Translating pre-OA prophylaxis into clinical practice requires concurrent early economic and ethical appraisals, targeted implementation strategies, prospective safety and pharmacovigilance registries, and proactive engagement with payers and regulators to mitigate the risks of unwarranted medicalization and inequitable uptake. Key ethical concerns include intervening in largely asymptomatic individuals without an established net benefit, opportunity costs and diversion of scarce resources, potential exacerbation of health disparities, and entrenched therapeutic nihilism—the belief that early intervention is futile—which may impede or retard appropriate translation. Accordingly, early development programs should follow a staged translational pathway: pilot mechanistic and biomarker-driven studies in well-characterized high-risk cohorts, parallel cost-effectiveness and value-of-information analyses to define decision thresholds, establishment of prospective safety registries and active pharmacovigilance, and early iterative regulatory and payer consultations preceding any broad prophylactic deployment. Embedding deprescribing algorithms, predefined stopping rules, and explicit equity metrics within study and implementation plans will help ensure that any future prophylactic strategy delivers demonstrable population benefits while minimizing harm.

### 11.4. Additional Ethical, Economic, and Societal Considerations in Early OA Prevention Strategies

The prospect of long-term prophylactic pharmacotherapy in asymptomatic at-risk individuals raises important ethical, economic, and societal challenges that must be addressed in parallel with mechanistic and clinical development. First, there is a substantial risk of medicalizing large population segments if broad, nonselective prophylaxis is pursued [291]. This risk strongly argues for targeted approaches (biomarker- or event-triggered therapy) restricted to high-probability progressors rather than universal treatment. Second, long-term safety and polypharmacy are major concerns. Chondroprotective agents intended for preventive use should demonstrate exceptionally favorable safety profiles, and trial programs must proactively evaluate interactions with common chronic medications, while specifying deprescribing algorithms and monitoring triggers [292,293]. Even agents with an established reputation for safety, such as glucosamine, illustrate this need for caution. Emerging data suggest possible associations with altered glucose metabolism and increased intraocular pressure, raising questions regarding their suitability for long-term prophylactic use in otherwise healthy individuals [266,294,295]. Third, cost-effectiveness and equity require early integration into translational planning; decision-analytic and budget-impact modelling should identify realistic use cases (e.g., temporally bounded post-traumatic cohorts) in which benefits plausibly outweigh costs [3,296,297,298]. Early engagement with regulators, payers, and patient representatives is essential to define acceptable surrogate endpoints, patient selection criteria, and reimbursement pathways that minimize perverse incentives and promote equitable access [299]. Finally, shared decision-making, transparent informed consent, and prospective post-marketing surveillance (including registries that capture deprescribing outcomes) should be embedded in the study design and implementation to mitigate over-medicalization while enabling careful evaluation of the population-level benefits and harms [292,300,301,302].

## 12. Limitations and Future Directions

Modern molecular technologies—single-cell and spatial transcriptomics, time-resolved proteomics, and matrisome profiling—offer unprecedented resolution of early, pre-clinical cartilage changes, yet important technical, biochemical, and practical constraints limit interpretation and clinical translation, particularly when attempting to combine molecular biomarkers with advanced imaging for pre-OA diagnosis. At the molecular level, single-cell and spatial transcriptomics remain susceptible to dissociation artifacts, dropout, and poor detection of low-abundance transcripts; proteomics faces sensitivity and dynamic-range limits and incomplete coverage of heavily cross-linked or insoluble ECM components; and matrisome measurements are confounded by extensive post-translational modifications and proteolytic processing that complicate accurate quantification and tissue assignment. Imaging modalities, including compositional and quantitative MRI (e.g., T2/T1ρ mapping, dGEMRIC), and emerging molecular imaging, often lack the biochemical sensitivity and spatial resolution of molecular assays, producing a persistent scale-and-sensitivity mismatch that complicates co-registration and mechanistic interpretation [84,303]. Biomarker specificity is further compromised because candidate circulating or synovial markers frequently reflect systemic turnover or contributions from multiple joint tissues rather than cartilage-confined and cell-autonomous pathology [4,14,15,26,79,80,304,305,306]. Practical barriers to clinical translation include the absence of standardized, validated assays and imaging protocols, variable pre-analytic handling, small and heterogeneous study cohorts, unclear diagnostic thresholds and undefined normative ranges, high costs and limited access to advanced imaging, and inter-reader variability in image interpretation. Importantly, the predominance of cross-sectional data limits the demonstration that combined molecular–imaging signatures prospectively predict the transition from reversible, stress-responsive pre-OA states to irreversible clinical OA. Addressing these gaps will require harmonized multicenter longitudinal studies with spatially matched sampling, standardized laboratory and imaging pipelines and quality-control metrics, validated clinically scalable assays and clear diagnostic thresholds, larger well-phenotyped/endotyped study populations, robust spatially aware multi-omic and imaging integration methods with rigorous co-registration procedures, and experimental validation (including causal perturbation and model systems) to establish whether adding molecular biomarkers to imaging improves early diagnosis and individual-centered outcomes.

While the “pre-OA” concept remains provisional, its feasibility is supported by emerging translational evidence. Sensitive compositional MRI techniques (T2/T1ρ, dGEMRIC) can detect subtle, potentially reversible biochemical cartilage abnormalities years before radiographic change, and longitudinal imaging trajectories have been associated with subsequent structural progression [84]. Concurrently, high-throughput proteomic and metabolomic studies have identified candidate stress-responsive molecular signatures that discriminate between early and progressive states, although these require external replication and analytical validation [85]. Targeted assays for aggrecan neo-epitopes (e.g., ARGS), matrix fragments (COMP, FN-fs), and cartilage structural proteins (HAPLN1, CRTAC1) are promising sensitive indicators of early matrix turnover. Previous imaging studies increasingly suggested that these early perturbations often follow temporally ordered trajectories that may define a putative window of reversibility prior to irreversible fibrillation and subchondral remodeling [69]. Emerging optical and spectroscopic modalities (Raman and near-/short-wave infrared, NIR/SWIR) provide proof-of-concept molecular fingerprinting that can complement imaging and fluid biomarkers in multimodal pipelines [23,25,47]. Translating these tools into a clinically useful pre-OA definition will require coordinated efforts to standardize imaging and biospecimen protocols; develop analytically validated, scalable assays; assemble sufficiently powered, sex- and age-stratified longitudinal cohorts with spatially co-registered sampling; and perform mechanistic perturbation studies and prospective validation to demonstrate that identified signatures mark reversible, at-risk states rather than transient or adaptive variation. Accordingly, the framework proposed here should be viewed as a translational scaffold—a guide for integrating molecular, imaging, and biomechanical metrics into harmonized multimodal validation studies, rather than as an immediately actionable diagnostic entity.

A promising complementary approach is the application of dynamic network methodologies to define pre-OA states. DNBs, originally developed in systems biology to detect imminent critical transitions in complex diseases, provide a formal dynamical-systems framework for identifying tipping points in high-dimensional molecular systems by detecting small modules that (1) show a marked increase in intra-module variance (fluctuation), (2) exhibit heightened internal correlation/coherence, and (3) concurrently decouple from the background network [29,30,31,36,94,307]. Conceptually, DNB may help integrate heterogeneous mechanical, biochemical, imaging, spectroscopic, and (epi)genetic signals into temporally or individually resolved network states that capture system instability preceding overt structural degeneration. Recent multi-omic studies in cartilage biology, such as integrated transcriptomics–metabolomics profiling of porcine chondrocyte development [307], have demonstrated the experimental feasibility of generating coordinated, time-resolved datasets required for network-level analyses. Importantly, DNBs have been successfully used in other bone-related contexts. For example, a recent study identified a “pre-osteoporosis” triggering phase in which candidate DNB members satisfied the three statistical conditions and predicted the critical transition to developing osteoporosis [308]. Extensions of this family of methods include the single-sample DNB (sDNB), which constructs an individual-level predictor from the same three statistical criteria applied to a single sample [31]. Interestingly, the sDNB is model-free, does not require training on large case/control cohorts, and therefore mitigates overfitting inherent to population-based predictors while enabling per-individual early-warning inference. However, translational application to human pre-OA faces major challenges. Specifically, DNB inference optimally requires temporally dense sampling or well-chosen surrogate measures in accessible biofluids or imaging, and standards for multimodal network construction and cross-platform normalization are immature. Additionally, putative DNB signatures require rigorous ground-truthing in model systems and small prospective human pilots to distinguish true pre-disease transitions from transient physiological variability (e.g., in puberty, pregnancy, and menopause). Nonetheless, integrating DNB frameworks with evolving noninvasive modalities (e.g., advanced Raman/NIR spectroscopy and compositional MRI) and adopting staged validation pipelines (phantom/cadaver, ex vivo/animal, invasive-validation human pilots, and larger longitudinal cohorts) may ultimately enable network-level early warning signals that complement conventional biomarkers [23,25,29,30,31,47]. Developing harmonized preprocessing and inference pipelines, conducting temporally resolved multi-omic and multimodal studies, implementing temporally dense sampling strategies in well-phenotyped high-risk cohorts, and performing robust cross-modal validation will be essential to determine whether DNB/sDNB-informed signatures can operationalize a system-level definition of pre-OA.

Although this study emphasizes a cartilage-centric endotype to illustrate dynamic concepts and actionable windows for ultra-early intervention, hyaline cartilage functions within an integrated joint organ: muscle, ligaments, menisci, synovium, subchondral bone, peri-articular fat, and the neurovascular milieu continuously interact with cartilage and can act as primary drivers, modulators, or secondary targets of joint pathology [93]. These organ-level couplings and systemic modifiers (metabolic dysregulation, immune activation, and hormonal transitions) substantially reshape the dynamical landscape, including the basin depth, saddle height, and barrier geometry, thereby altering the transition probability and reversibility (see Figure 1, for details). Joint tissues also follow lifespan-dependent, sex-specific, and context-dependent trajectories (e.g., puberty, pregnancy, and menopause) that often produce tissue variations without inevitable progression to OA. Translating the landscape metaphor into clinically actionable pre-OA criteria therefore demands (i) explicit modelling of organ-level couplings and sex/age modifiers in computational frameworks, (ii) spatiotemporally (stochastic) resolved longitudinal cohorts that enable co-registration of imaging with tissue and fluid biomarkers across life stages and perievent windows; and (iii) experimental perturbation studies (ex vivo human osteochondral cultures, targeted animal models, and mechanobiological manipulations) to test causality and quantify multi-tissue effects on system resilience. Practically, cartilage-level signals (compositional MRI, matrix-derived biomarkers) should initially be treated as proxies for local attractor states until they are validated within multimodal, organ-aware signatures. Clinical operationalization of “pre-OA” will necessarily require harmonized, validated multimodal thresholds that discriminate adaptive, transient tissue changes from true pre-disease states. Parallel efforts must harmonize and operationalize multimodal biomarker panels and diagnostic thresholds, develop scalable assays and imaging protocols, and embed these tools within prospective interventional studies that evaluate whether restoring resilience (deepening healthy basins, raising saddles) reduces the progression to symptomatic, structurally evident OA. Only through coordinated computational, experimental, and longitudinal translational programs can the heuristic landscape be robustly extended to organ-level, lifespan-aware models suitable for clinical decision-making.

An important and underappreciated source of biological heterogeneity relevant to defining pre-OA arises from the dynamic influence of sex hormones throughout life. Circulating sex steroids (estrogens, progesterone, and androgens), transient reproductive states (menstrual cycle phases, pregnancy, and lactation), exogenous hormonal exposure (combined and progestin-only contraceptives and menopausal hormone therapy), and menopausal transition modulate cartilage metabolism, subchondral bone remodeling, synovial biology, ligamentous laxity, and neuromuscular control. These effects create temporally structured windows of altered susceptibility and resilience: menstrual-cycle–related fluctuations can transiently change joint laxity and inflammatory mediators; pregnancy produces systemic and local biomechanical and hormonal adaptations; and menopause is associated with increased OA incidence and shifts in catabolic–anabolic balance. Prior studies have emphasized that these hormonal transitions interact with sex-specific differences in immune responses, cartilage metabolism, and ligamentous laxity, contributing to the marked increase in OA risk among postmenopausal women and reinforcing the need for sex-specific risk stratification [309,310,311,312]. Peshkova et al. [310] further highlight that anatomical, hormonal, molecular, and biomechanical sex-differences converge after menopause, including ligament remodeling, enhanced inflammatory responsiveness, and steep declines in sex-steroid levels, potentially accelerating OA onset and progression. The recent Global Burden of Disease analysis (1990–2021), with projections to 2035, documents a rising global and regional burden of OA and forecasts increasing prevalence and years lived with disability in aging populations and selected regions, thereby underscoring the urgent need for public health initiatives to establish sex- and life-stage–stratified longitudinal cohorts, harmonized biomarker pipelines, and biomarker-guided preventive and surveillance programs to address this growing challenge [312]. Consequently, female life-course variation introduces important sources of biological heterogeneity and complicates the interpretation of single time-point biomarkers and development of universal pre-OA thresholds. To address these complexities, future translational programs should: (i) systematically capture reproductive and hormonal variables (menstrual phase or cycle day, serum estradiol/progesterone/testosterone, where feasible, parity and timing of pregnancies, lactation history, age at natural or surgical menopause, and current/past hormonal therapies, including type and dose); (ii) implement cycle-aware or time-standardized biospecimen collection, record sampling time, and prespecify sex- and life-stage-stratified analyses and normative ranges; (iii) employ translational models (ex vivo and in vivo) that recapitulate defined hormonal states to test causality and validate sex-specific biomarkers and interventions; and (iv) design adaptive trials and analytic pipelines that permit the evaluation of hormone-sensitive timing and combined hormone/chronobiologic strategies. Establishing sex- and life-stage-specific normative ranges and biomarker thresholds are essential to ensure that pre-OA definitions and preventive programs are biologically informed, equitable, and clinically applicable across diverse life courses.

As mentioned earlier, the translational pathway from mechanistic insights to prophylactic interventions raises important ethical, economic, and implementation challenges that must be addressed explicitly in future programmes. Long-term pharmacological prevention in largely asymptomatic at-risk individuals risks medicalizing broad population segments, imposing substantial costs, and exposing otherwise healthy people to cumulative safety and polypharmacy burdens unless interventions are narrowly targeted and supported by robust evidence [291,293]. Crucially, neglecting the mechanical environment of the diarthrodial joint (e.g., abnormal loading or malalignment) can negate pharmacologic chondroprotection [313]. These biomechanical drivers help explain why many putative chondroprotective agents have not provided durable benefits when tested in isolation. Therefore, development strategies should either focus on populations in which biomechanical correction is feasible (e.g., well-characterized post-traumatic cohorts) or intentionally combine biological and biomechanical interventions [265,267]. To mitigate translational risk, I recommend the explicit integration of (i) early health-economic modelling and budget-impact assessment to identify realistic target populations and use cases [296]; (ii) implementation science and stakeholder engagement—including clinicians, researchers, patients, regulators, payers, health economists, and industry representatives—to define acceptable surrogate endpoints, establish reimbursement pathways, and ensure broad translational feasibility [299]; and (iii) prospective safety registries with predefined deprescribing algorithms to monitor long-term harms and interaction risks [292]. Therefore, these ethical and practical constraints become core research priorities: validating high-specificity multimodal biomarkers to avoid overtreatment, developing targeted trigger-based prophylaxis strategies, quantifying real-world cost-effectiveness, and designing trials that capture both clinical benefits and population-level harms. Addressing these limitations will require embedding health-economic, ethical, and implementation endpoints within mechanistic and clinical studies, rather than treating them as optional add-ons, to ensure that any future prophylactic strategy delivers a net population benefit.

## Figures and Tables

**Table 1:** Operational definition of OA stages integrating pathophysiology, clinical features and diagnostic criteria. Data adapted from references [4,14,15,18,20,21,23,25,26,35,44,46,69,70,71,72,73,74,75,76,77,78,79,80,81,82,83,84,85]. Abbreviations: AGEs, advanced glycation end products; ARGS-aggrecan, aggrecanase-derived aggrecan fragments; BML, bone marrow lesion; C1,2C, collagen type I/II cleavage products; Coll2-1, type II collagen neoepitope; COMP, cartilage oligomeric matrix protein; CPII, C-propeptide of type II procollagen; CRTAC1, cartilage acidic protein 1; CTX-II, C-terminal telopeptide of type II collagen; dGEMRIC, delayed gadolinium-enhanced magnetic resonance imaging of cartilage; hsCRP, high-sensitivity C-reactive protein; IL, interleukin; JSN, joint space narrowing; KL, Kellgren–Lawrence; MMP, matrix metalloproteinase; MRI, magnetic resonance imaging; ROM, range of motion; TNF, tumor necrosis factor; WOMAC, Western Ontario and McMaster Universities Osteoarthritis Index.

Stage	Characteristics	Clinical Manifestations	Diagnostic Criteria
Pre-OA	No structural damage; at-risk joint with early molecular or biomechanical perturbations; imbalance in anabolic/catabolic signaling; metabolic dysregulation in predisposed individuals (injury, obesity, genetic or epigenetic factors)	No clinical signs; occasional joint sensitivity; risk stratification based on molecular biomarkers and imaging	Relative elevations in early turnover serum or synovial biomarkers (e.g., COMP, ARGS-aggrecan and hyaluronic acid fragments, CRTAC1 ^1^) or low-grade inflammatory signals compared with controls (increased activity/expression of selected ILs and MMPs), indicating net catabolic balance; elevated leptin and adiponectin in obese individuals. Normal radiographs (KL 0); MRI T2 mapping showing altered cartilage hydration; dGEMRIC signal alteration without focal cartilage defect. Subtle gait changes or altered joint loading in at-risk groups (e.g., post-ACL injury, obesity-related overload).
Early OA	Initial cartilage breakdown; collagen disorganization and softening; emergence of SASP, chondrocyte hypertrophy, and apoptosis; low-grade inflammation; minor or often undetectable changes on imaging; minimal morphologic change	Asymptomatic or intermittent, activity-related pain or stiffness; no objective joint swelling or crepitus; no or minimal functional impairment; no effusion	Slightly elevated catabolic markers (sHA, sCOMP, and sMMP-3, Coll2-, uCTX-II, CPII, C2C, C1,2C); early inflammatory cytokines (TNF-α, IL-1β, IL-6) in synovial fluid/serum; altered cartilage methylation profiles. Normal radiographs (no JSN or osteophytes) or equivocal (KL 0–1); dGEMRIC/T1ρ signal change; MRI showing superficial fibrillation, focal cartilage softening. Abnormal gait kinetics; reduced shock absorption; asymmetrical loading pattern; subtle decline in muscle strength or proprioception.
Mild OA	Focal cartilage thinning; JSN visible on radiographs; early subchondral bone remodeling; small osteophytes	Activity-related joint discomfort; transient morning stiffness (<30 min); slight functional limitations	Radiographic KL 1–2 with focal JSN/osteophytes; MRI showing focal cartilage defect, small-to-moderate BMLs; ultrasound showing synovial thickening absent or mild. Elevated cartilage degradation markers (COMP, CTX-II), altered CPII/C2C ratios indicating increased catabolism; synovial cytokine profile variable. Minor decrease in walking speed or joint stability; quadriceps weakness, altered gait patterns.
Moderate OA	Marked cartilage loss; osteophyte enlargement; subchondral sclerosis; cartilage fissuring or partial thickness loss; synovial hypertrophy; synovitis	Persistent pain; crepitus; intermittent effusion; functional decline; reduced ROM	KL grade 2–3 with definite JSN, evident osteophytes, sclerosis; MRI showing full-thickness focal defects, larger BMLs, synovitis on contrast-enhanced sequences. High levels of matrix degradation markers (MMP-13, CTX-II, AGEs), increased inflammatory mediators (IL-6); synovial fluid shows inflammatory cell increases. Reduced loading tolerance; gait asymmetry; quadriceps weakness; reduced 6-min walk distance; decline in timed-up-and-go; WOMAC function score ≥40.
Severe OA	Near-complete cartilage loss or complete erosion; bone-on-bone contact; large osteophytes; joint deformity and fibrosis; synovial thickening and persistent inflammation	Constant pain (including at rest/night); marked stiffness; swelling; instability/deformity; severe functional limitation; disability	KL grade 3–4; marked JSN; large osteophytes; subchondral cysts; MRI showing diffuse cartilage loss and large BMLs; osteophytes; joint deformity; synovial hypertrophy/fibrosis. Plateau or decline in cartilage turnover markers with persistent systemic inflammation (hsCRP, IL-6); elevated N-α-acetyl-L-asparagine ^2^ as a metabolic marker of advanced OA; high viscosity changes, abundant inflammatory cells, and increased catabolic enzyme activity. Severe mobility limitation, need for assistive devices, or surgical indication (joint replacement candidate).

^1^ Cartilage acidic protein 1 (CRTAC1) has recently emerged as a promising biomarker capable of discerning subtle changes in the structure of glycosaminoglycans within the ECM of the knee cartilage through plasma proteomic analysis [46]. ^2^ N-α-acetyl-L-asparagine has been reported as a metabolic biomarker associated with late-stage OA, reflecting disrupted amino acid metabolism and chronic inflammatory burden [78].

**Table 2:** Representative molecular classes involved in cartilage degeneration from pre-OA through disease chronification, structural joint damage, and the emergence of clinical symptoms. These early, potentially reversible and measurable changes are candidate diagnostic biomarkers and therapeutic targets for OA pathogenesis. Data from references [11,12,13,146,148,149,162,170,171,172,173,174,175,176,177,178,179,180,181,182].

Key Activators	Primary Receptor	Major Signalling Pathways	Principal Downstream Effects
ECM fragments (MDPs)—e.g., FN-fs, COMP, aggrecan ARGS peptides, LMW-HA, tenascin-C	Integrins (α5β1, etc.), CD44, TLR2/TLR4 (and other innate sensors)	NF-κB; MAPKs (p38, ERK, JNK); can promote NLRP3 inflammasome	Induction of pro-inflammatory cytokines and chemokines; upregulation of MMPs/ADAMTS; synovial inflammation, chondrocyte catabolic shift, feed-forward ECM degradation and pain sensitization
Pro-inflammatory cytokines—e.g., IL-1β, TNF-α, IL-6 (classical & trans-signalling)	IL-1R1 (+IL-1RAcP); TNFR1/2; IL-6R/gp130 (±sIL-6R)	MyD88 → NF-κB; MAPK; JAK/STAT3 (IL-6); AP-1	Strong induction of proteases (MMPs, ADAMTS), suppression of ECM biosynthesis (aggrecan, collagen II), chondrocyte hypertrophy/apoptosis, synovitis and bone remodeling
Matrix-degrading proteases—e.g., ADAMTS-5, MMP-13, cathepsin K	Secreted enzymes (their cleavage products engage CD44, TLRs, integrins on neighboring cells)	Proteolytic generation of DAMPs → secondary activation of NF-κB/MAPK and downstream transcriptional programs	Proteolysis of aggrecan and collagen → loss of cartilage biomechanical integrity, irreversible collagen network breakdown, progression of structural OA and amplification of inflammatory/proteolytic loops

**Table 3:** Core cartilage clock genes and concise summary of effects observed after clock disruption or targeted manipulation (representative model systems and primary references cited). Data from references [209,210,211,212,213,214,215,216,217,218,219,220,221].

Gene	Role (Anabolic/Catabolic/Clock/Signalling)	Effect of Clock Disruption or Manipulation	Model Sample/Reference
BMAL1 (ARNTL)	Clock/pro-homeostasis	Chondrocyte-specific Bmal1 ablation abolishes local cartilage circadian rhythms and reduces anabolic markers (SOX9, ACAN, COL2A1) while impairing TGF-β signaling (reduced p-SMAD2/3) and upregulates catabolic proteases (MMP1, MMP3, MMP13, MMP14, ADAMTS5, ADAMTS5), shifting chondrocytes into a catabolic, repair-deficient state that leads to progressive cartilage degeneration	Chondrocyte-specific Bmal1 KO (Col2a1-Cre; Bmal1), PER2::LUC cartilage; mouse and human cartilage [211,212,213,214,217]
CLOCK	Clock (activator with BMAL1)	Perturbation of the CLOCK:BMAL1 transcriptional axis dampens rhythmic expression of cartilage matrix and metabolic genes and shifts transcriptional programmes toward catabolic profiles	Murine cartilage time-series and cultured chondrocytes; circadian transcriptomics [209]
PER1	Clock (negative limb/signalling)	Rhythm disruption or altered PER1 expression associates with increased inflammatory sensitivity and higher expression of matrix-degrading enzymes in cartilage/rhythm-disruption models	Primary chondrocytes, cartilage explants and in vivo rhythm-disruption (jet-lag/inflammatory stimuli) models [209,223]
PER2	Clock (negative limb/reporter)	PER2::Luc reports robust cartilage rhythms that are dampened with ageing/OA; PER2 loss/dampening reduces protective rhythmic responses and modifies inflammatory/matrix outcomes	PER2::LUC reporter cartilage, primary chondrocytes (mouse/human); time-series bioluminescence and gene expression [209]
CRY1	Clock (negative limb)	Reduced CRY1 amplitude reported in OA/damaged cartilage; decreased CRY1 correlates with heightened catabolic/inflammatory gene induction	Human OA chondrocytes, mouse cartilage (expression profiling/KO analyses) [215]
CRY2	Clock (negative limb)	CRY2 expression/amplitude is reduced in OA; Cry2 deficiency increases OA severity in experimental models and is associated with upregulated MMP/ADAMTS catabolic programmes	Human OA samples and Cry2 KO mouse OA models; RNA-seq and histopathology [215]
NR1D1 (REV-ERBα)	Clock/signalling (repressor)	NR1D1/REV-ERBα downregulation in aging/OA cartilage; genetic or pharmacologic perturbation modifies TGF-β signalling and matrix gene expression and alters mechanotransduction responses	Human chondrocyte cultures, cartilage explants and OA models; knockdown/agonist studies [220,221]
RORα (RORA)	Clock/transcriptional regulator	RORα contributes to transcriptional control of inflammatory and metabolic targets in chondrocytes; modulation of RORα changes cytokine responsiveness and downstream catabolic signalling	In vitro human chondrocytes and experimental cartilage injury models; targeted perturbation studies [216]
BHLHE40 (DEC1)/BHLHE41 (DEC2)	Clock/signalling (repressors & stress-responsive)	Stress/inflammatory upregulation of DEC1/DEC2 perturbs clock outputs in chondrocytes and promotes pro-catabolic signalling; manipulation alters rhythmic matrix/inflammatory gene expression	Primary chondrocytes under inflammatory or mechanical stress; cartilage injury models and circadian studies [216,219]

**Table 4:** Structural and functional comparison of pericellular, territorial, and interterritorial ECM zones in articular cartilage: key molecular components and their mechanical and biological functions. Data adapted from references [48,49,232].

ECM Cartilage	Location/Appearance	Major Components (Representative)	Primary Function/Mechanical Role
Pericellular	Immediately surrounds individual chondrocytes (chondron); thin capsule often highlighted by type VI collagen immunostain	Type VI collagen; perlecan (heparan sulfate proteoglycan); small leucine-rich proteoglycans (decorin, biglycan); hyaluronan; multi-adhesive link proteins or hyaluronan- and proteoglycan-binding proteins (e.g., HAPLN1)	Specialized pericellular microenvironment for mechanotransduction and cell–matrix signaling; attenuates cell-level strains and mediates biochemical exchange
Territorial	Encircles the pericellular matrix as an intensely basophilic “rim” (basket-like) around single cells or cell clusters	High local aggrecan concentration (proteoglycan-rich); type II collagen present in dense/disorganized bundles; hyaluronan; link proteins	Local compressive buffering and high fixed-charge density near cells; mechanical shielding of chondrons and modulation of ion/water movement
Interterritorial	Bulk ECM between chondrons; less basophilic than the territorial rim; shows organized collagen fibrils under polarized light	Type II collagen fibrillar network (major bulk collagen); aggrecan aggregates bound to hyaluronan; high tissue hydration	Primary tissue-level load bearing—tensile strength from collagen network and compressive resistance/resilience from proteoglycans and water

**Table 5:** Phase-resolved proteomic map of cartilage across a 24-h cycle. Representative rhythmically abundant proteins are grouped by dominant pathway and peak phase. Adapted from [217].

Phase	Peak Pathway (s)	Representative Molecules	Cartilage Function
Morning (early rest)	Cytoskeletal remodelling; proteasomal degradation	PSMB7, PSMD2, PSMD5; PLOD1/2 (collagen cross-linking enzymes); SERPINE1 (protease inhibitor)	Supports structural rearrangements; proteasomal turnover removes damaged proteins, while PLOD-mediated cross-linking and SERPINE1 activity stabilize collagens and prevent premature degradation, enhancing tensile strength
Afternoon (late rest)	Protein synthesis (e.g., ribosomal function)	RPL5, RPL23a, RPS3a1	Upregulated translation machinery supports de novo synthesis of matrix components (e.g., glycoproteins)
Evening (early active)	ATP synthesis (energy metabolism)	HSPA9, HSP90AA1, HSP90AB1; MATN1; SERPINE1; PLOD2	Chondrocytes increase glycolysis and oxidative phosphorylation to meet energy demands
Night (late active)	Glucose metabolism; proteostasis	SLC2A1 (GLUT1); PKM (pyruvate kinase); protein synthesis (e.g., molecular chaperones)	Increased glucose uptake, while chaperone expression support mechanical resilience

## Data Availability

No new data were created or analyzed in this study. Data sharing is not applicable to this article.

## Abbreviations

The following abbreviations are used in this manuscript:

AAOS	American Academy of Orthopaedic Surgeons
ACAN	Aggrecan
ADAMTS	A disintegrin and metalloproteinase with thrombospondin motifs
AMPK	AMP-activated protein kinase
ARGS	Aggrecanase-generated N-terminal neoepitope
ATP	Adenosine triphosphate
BHLHE40	Basic helix–loop–helix family member e40
BMAL1 (ARNTL)	Brain and muscle ARNT-like 1
BML	Bone marrow lesion
CADENCE	Chondroprotection Advanced through Deliberate Exercise and Networked Circadian Engagement
CD44	Cluster of differentiation 44
cKO	Conditional knockout (tissue- or time-specific gene deletion)
CLOCK	Circadian locomotor output cycles kaput
COL2A1	Type II collagen alpha-1 chain
COMP	Cartilage oligomeric matrix protein
CRY	Cryptochrome proteins
CS	Chondroitin sulfate
CTX-II	Cross-linked C-telopeptide fragments of type II collagen
DAMPs	Damage-associated molecular patterns
DMOADs	Disease-modifying osteoarthritis drugs
DNA	Deoxyribonucleic acid
DNBs	Dynamic Network Biomarkers
ECM	Extracellular matrix
ELISA	Enzyme-Linked Immunosorbent Assay
EMG	Electromyography
ERK	Extracellular signal-regulated kinase
FN	Fibronectin
Fn-fs	Fibronectin fragments
Glc	Glucose
GS	Glucosamine sulfate
HbA1c	Glycated haemoglobin
HA	Hyaluronic acid
HAPLN1	Hyaluronan and proteoglycan link protein 1
HELIX-II	Type-II collagen helical peptide
HK	Hexokinase
HSPA9	Heat shock protein family A member 9 (mortalin)
HSP90 (HSP90AA1/HSP90AB1)	Heat shock protein 90 family
HST	Heel-strike transient
IGF-1	Insulin-like growth factor 1
IL-1β	Interleukin-1 beta
IL-1RAcP	Interleukin-1 receptor accessory protein
iNOS	Inducible nitric oxide synthase
iPSC	Induced pluripotent stem cells
JAK/STAT3	Janus kinase/signal transducer and activator of transcription 3
JSN	Joint space narrowing
JSW	Joint space width
KS-5D4	KS-5D4 (biomarker/assay designation)
LMW-HA	Low-molecular-weight hyaluronan
L/P ratio	Lactate/pyruvat ratio
MAPK	Mitogen-activated protein kinase
MATN1	Matrilin-1
MDPs	Matrix-derived degradation products
MMPs	Matrix metalloproteinases
MRI	Magnetic resonance imaging
MSM	Methylsulfonylmethane
mRNA	Messenger ribonucleic acid
MyD88	Myeloid differentiation primary response 88
NAC	N-acetylcysteine
NADH	Nicotinamide adenine dinucleotide
NF-κB	Nuclear factor kappa-light-chain-enhancer of activated B cells
NFATC2	Nuclear factor of activated T-cells
NIR	Near-Infrared
NLRP3	NOD-, LRR- and pyrin domain-containing protein 3 inflammasome
NR1D1 (REV-ERBα)	Nuclear receptor subfamily 1 group D member 1
OA	Osteoarthritis
OARSI	Osteoarthritis Research Society International
OXPHOS	Oxidative phosphorylation
PAPS	3′-phosphoadenosine-5′-phosphosulfate
PER	Period circadian proteins
PER2::LUC	PER2 luciferase reporter
sPIIANP	Procollagen type IIA N-terminal propeptide (serum PIIANP)
PRG4	Proteoglycan 4
PKM	Pyruvate kinase M
PLOD	Procollagen-lysine, 2-oxoglutarate 5-dioxygenase
PRP	Platelet-rich plasma
PRR	Pattern recognition receptor
PSMB/PSMD	Proteasome subunits B and D family
PTOA	Post-traumatic osteoarthritis
pre-OA	pre-osteoarthritis
RA	Rheumatoid arthritis
RNA	Ribonucleic acid
RNS	Reactive nitrogen species
RPL	Ribosomal protein
ROI	Regions of interest
ROM	Range of motion
RORA (RORα)	Retinoic acid-related orphan receptor alpha
ROS	Reactive oxygen species
SASP	Senescence-associated secretory phenotype
SCN	Suprachiasmatic nucleus
sDNB	single-sample Dynamic Network Biomarker
SERPINE1	Serpin family E member 1 (plasminogen activator inhibitor-1, PAI-1)
SCS	Stagnant cartilage syndrome
SLC2A1 (GLUT1)	Solute carrier family 2 member 1 (glucose transporter 1, GLUT1)
SOX9	SRY-box transcription factor 9
SWIR	Short-wave infrared
TCA cycle	Tricarboxylic acid (Krebs) cycle
TGF-β	Transforming growth factor-beta
TIMP	Tissue inhibitor of metalloproteinases
TLR	Toll-like receptor
TNF-α	Tumor necrosis factor-alpha
TNFR1/2	Tumor necrosis factor receptors 1 and 2
UTE	Ultrashort echo time
vGRF	Vertical ground reaction force
